# Antimicrobial Activity of Polyphenols and Alkaloids in Middle Eastern Plants

**DOI:** 10.3389/fmicb.2019.00911

**Published:** 2019-05-15

**Authors:** Leen Othman, Ahmad Sleiman, Roula M. Abdel-Massih

**Affiliations:** ^1^Faculty of Medicine, University of Balamand, El-Koura, Lebanon; ^2^Department of Biology, University of Balamand, El-Koura, Lebanon

**Keywords:** antimicrobial resistance, polyphenols, alkaloids, plant secondary metabolites, resistance-modifying agent

## Abstract

Antibiotic-resistant microorganisms have been an ever-growing concern over the past years. This has led researchers to direct their attention onto plants to be able to discover new possible antimicrobial compounds. The Middle East encompasses a wide spectrum of plant diversity with over 20,000 different species in habitats ranging from deserts to snow-capped mountains. Several plant secondary metabolites and their derivatives have been identified as possible antimicrobial agents. Among the secondary metabolites studied, alkaloids and polyphenols have shown strong antimicrobial activity. Polyphenols are one of the most numerous and diverse group of secondary metabolites; their antioxidant properties provide the basis for antimicrobial effects. Alkaloids provided the underlying structure for the development of several antibiotics with a diverse range of action. The ability of some plant secondary metabolites to act as resistance-modifying agents is a promising field in mitigating the spread of bacterial resistance.

## Introduction

The search for remedy in plants is not new. The limited effective life span of current antibiotics, the lack of compliance of patients, the unmonitored use in agriculture, and the slow rate in releasing new antimicrobial agents have led to an alarming increase in antimicrobial resistance. Multidrug-resistant (MDR) microorganisms cause almost 50% of the worldwide hospital-acquired infections. Among those, a few strains are of particular concern such as bacteria producing expanded spectrum beta-lactamases (ESBL) like *Escherichia coli, Pseudomonas aeruginosa, Klebsiella pneumoniae, Acinetobacter baumanii*, and *Helicobacter pylori*, in addition to vancomycin-resistant *Enterococci* (VRE) as well as *Mycobacterium tuberculosis*, penicillin-resistant *Streptococcus pneumoniae, Shigella*, and *Salmonella* (Sibanda and Okoh, 2007; Abreu et al., 2012). Scientists, even clinicians, around the world are interested in searching for new bioactive components in plants.

Moreover, the public is currently more interested in switching toward natural remedies. Another driving factor in the interest in discovering plant antimicrobials is the rapid extinction (due to climate change and human activity) of plants that may lead to the loss of potentially active plant components.

There is a wide range of plant species on Earth (400,000–500,000 species). Plants have a great ability to produce secondary metabolites such as phenolics and polyphenols, alkaloids, terpenoids and essential oils, lectins, and others. In this review, we will mainly focus on the antimicrobial activity of polyphenols and alkaloids in plants studied in the Middle East area and will give an overview of some medicinal plant extracts studied in the region.

## Middle Eastern Plant Extracts with Antimicrobial Activity

Middle Eastern plants were traditionally used as sources for oral hygiene in ancient times. *Salvadora persica* L. or miswak chewing sticks are widely used in the Arab world and more specifically Islamic cultures as a source of oral hygiene. It has a wide geographical distribution over the Middle East and most of the African countries. Miswak, or the arak tree, was analyzed using the disc diffusion and micro-well dilution assays. Aqueous extracts exhibit antimicrobial properties against seven microbial species including *Streptococcus mutans, Streptococcus faecalis, Streptococcus pyogenes, Lactobacillus acidophilus, P. aeruginosa, Staphylococcus aureus*, and *Candida albicans*. *Streptococcus* species are the most sensitive, with the highest activity observed against *S. faecalis* (inhibition zone, 22.3 mm), and the most resistant was *P. aeruginosa* (Al-Bayati and Sulaiman, 2008). Similarly, crude extracts of miswak were tested against *Porphyromonas gingivalis, Prevotella intermedia, Actinobacillus actinomycetemcomitans, Actinomyces naeslundii*, and *C. albicans*. Ethanolic extracts demonstrated the strongest antimicrobial activity; extracts of the root were more inhibitory than those of the twig. Minimum inhibitory concentration (MIC) ranged between 100 and 300 mg/ml for different crude extracts (AbdElRahman et al., 2002). In another study, the antibacterial activity of the methanolic and aqueous extracts of *S. persica* was tested against 45 bacterial strains of *S. aureus, S. mutans, L. acidophilus*, and *P. aeruginosa* (Sher et al., 2011). Miswak contains several secondary metabolites, such as alkaloids, saponins, volatile oils, terpenoids, flavonoids, and carbohydrates. The MIC results showed that the aqueous extract of *S. persica* had better antibacterial activity than the methanolic extract against all the tested strains. Its best activity was against *S. aureus*, with a MIC value of 2.49 mg/ml, and its lowest activity was against *P. aeruginosa*, with a MIC value of 7.34 mg/ml (Sher et al., 2011). Similarly, the diameters of the zone of inhibition ranged from 12.6 to 22.3 mm for the aqueous extract and from 9.2 to 15.7 mm for the methanolic extract using the agar diffusion method (Sher et al., 2011).

A wide variety of plants tested from various parts of the Middle East demonstrate antimicrobial activity. Shahat et al. (2017) studied the antimicrobial activity of methanolic extracts from 24 Saudi Arabian herbal medicinal plants. Most of the plants demonstrated activity against at least two of the eight microorganisms tested (*K. pneumoniae, Proteus vulgaris, P. aeruginosa, Serratia marcescens, Bacillus cereus, Micrococcus luteus, Micrococcus roseus*, and *S. aureus*). *Echium arabicum, Rhantarium epapposum, Rumex vesicarus, Ziziphus nummularia, Caylusea hexagyna*, and *Artemisia monosperma* had antimicrobial activity against most of the tested microbial species (Shahat et al., 2017). Their antimicrobial activity against bacteria was more effective than against fungi. *Z. nummularia* exhibited the highest antibacterial activity against all tested bacteria; *E. arabicum* extract displayed activity against all tested bacteria except for *B. cereus*.

Similarly, extracts from five indigenous plants, *Rosmarinus officinalis, Pisidium guajava, Punica granatum* peel, *Vitis vinifera*, and *Teucrium polium*, used as medicinal plants in Palestine, were studied for their antimicrobial activity. *R. officinalis* demonstrated the highest antimicrobial activity against the tested microbial species (*S. aureus, E. coli, P. aeruginosa, K. pneumoniae, Bacillus subtilis, M. luteus, C. albicans*, and *Aspergillus niger*; Qabaha, 2013). *Rheum rhaponticum* (false rhubarb), *Olea europaea, Viola odorata, Rosmarinus officinalis, Origanum majorana*, and *Trigonella foenum-graecum*, plants indigenous to Lebanon, were tested on Extended Spectrum Beta Lactamase (ESBL) producing *E. coli* and *K. pneumoniae*. The plants were extracted with ethanol before further sub-fractionation with different solvents. The majority of the tested strains had an MIC range between 2.5 and 80 μg/ml (Abdel-Massih et al., 2010; Daoud et al., 2011).

Other plants in the region with similar antibacterial activity are the wild Lebanese *Satureja cuneifolia, Coridothymus capitatus, Satureja thymbra, Thymus syriacus*, and *Thymbra spicata*. They were analyzed against *C. albicans* and six bacterial strains including *E. coli, S. aureus, Enterococcus faecalis, Salmonella enteritidis, P. aeruginosa*, and *B. subtilis*. The essential oil extracts of *C. capitatus* demonstrated an inhibitory effect on all tested strains with an MIC ranging between 0.2 and 0.8 mg/ml. The MICs for *T. spicata, S. thymbra*, and *T. syriacus* ranged between 0.2 and 0.6 mg/ml, between 0.1 and 0.4 mg/ml, and between 0.64 and 0.8 mg/ml, respectively. The strongest inhibitory effect was observed with *S. thymbra* extract against *E. coli, E. faecalis*, and *B. subtilis*. Phytochemical screening demonstrated that these species were characterized by containing either thymol (54.3%) or carvacrol (60.9%; Al Hafi et al., 2017). The essential oils from six Lebanese conifers including *Abies cilicica, Cupressus sempervirens, Juniperus oxycedrus, Juniperus excelsa, Cedrus libani*, and *Cupressus macrocarpa* were tested against microorganisms responsible for human cutaneous infections. *J. oxycedrus, J. excelsa*, and *C. libani* demonstrated significant antibacterial activity against *S. aureus* with an MIC of 64 μg/ml. *Trichopyton* species including *T. mentagrophytes, T. soudanense, T. violaceum*, and *T. tonsurans* were sensitive to most plant extracts with MICs ranging between 32 and 512 μg/ml (Fahed et al., 2017).

Phytochemical screening of plants from Soqotra Island in Yemen exhibited antimicrobial activity mostly against Gram-positive bacteria with methanolic extracts demonstrating the highest activity. *Boswellia socotrana* exhibited the strongest antimicrobial activity with both methanolic and aqueous extracts. MICs ranged between 125 and >1,000 μg/ml (Mothana et al., 2009).

Plants *Mercurialis annua* L., *Bongardia chrysogonum* L., and *Viscum cruciatum* Sieb, used traditionally by herbalists in Jordan, were tested for their antimicrobial activity. An antimicrobial effect was only noticed with *V. cruciatum* extract against *S. aureus* (MIC 1.25), *C. albicans* (MIC 1.25), and *Propionibacterium acne* (MIC 0.625), but not *P. aeruginosa* (Assaf et al., 2013). Moreover, Jordanian plants *P. granatum* L., *Quercus infectoria* olive, and *Rhus coriaria* L. displayed antibacterial activity against 14 bacterial species including both Gram-negative and Gram-positive strains. Minimum inhibitory concentrations ranged between 4 and 32 mg/ml while Minimum bactericidal concentrations were between 8 and 62 mg/ml (Nimri et al., 1999).

*R. coriaria*, Syrian sumac plant, displayed MICs between 1 and 3.5 mg/ml against Gram-negative bacteria and MICs from 0.5 to 1.5 mg/ml against Gram-positive bacteria. MICs for yeast ranged between 5.2 and 7 mg/ml. The highest activity was against *B. cereus* and *H. pylori* with MICs of 0.5 and 1 mg/ml, respectively (Kossah et al., 2013; Al-Boushi et al., 2014). Similarly, *T. syriacus* extracts demonstrated antibacterial activity against Gram-negative isolates with MICs between 3.125 and 12.5 μg/ml. The main active compounds were thymol, carvacrol, dihydro-carvon, and linalool, respectively (Al-Mariri et al., 2013). *Q. infectoria* (Aleppo oak) aqueous extracts were most potent against *B. cereus* with an inhibition zone of 7.25 mm followed by *Yersinia enterocolitica* with an inhibition zone of 14.45 mm, while ethanolic extracts demonstrated 16.85- and 14.15-mm inhibition zones for *B. cereus* and *Y. enterocolitica*, respectively. Essential oil, ethanolic, and aqueous extracts had MICs of 0.064, 0.512, and 0.256 μg/ml, respectively, against *B. cereus* (Shariatifar et al., 2014).

Many of these plant extracts are rich in polyphenols and other secondary metabolites that can contribute to different biological activities. Polyphenols are known to have an anti-tumor effect, the ability to modulate the biological response by supporting the immune system and to protect cells from oxidative damage. Some of the protective mechanisms exhibited by polyphenols include induction of apoptosis, detoxification of xenobiotics, anti-oxidation, stimulation of the immune system, and anti-inflammatory properties. A possible mechanism of action of polyphenols is their effects on nuclear factors (NF-kB or activator protein-1) that are central molecules in the pathogenesis of cancer. Popular polyphenols with anticancer activity include quercetin, resveratrol, and epigallotachein-3-gallate present in green tea and curcumin (Niedzwiecki et al., 2016).

Many indigenous or endemic plants were studied in the Middle East for antimicrobial activity, but the focus of this review will be mainly on the antimicrobial activity of polyphenols and alkaloids extracted from these plants.

## Groups of Antimicrobial Secondary Metabolites

Isolated secondary metabolites are estimated to be <10% of the total number available in plants. These metabolites are generally used as defense mechanisms against insects, herbivores, and microorganisms. The wide variety comes from the plants' ability to synthesize a large arsenal of aromatic compounds and their oxygen-substituted derivatives (Cowan, 1999).

For instance, the native Middle Eastern plant *Salvia officinalis* (sage) includes a wide spectrum of plant secondary metabolites. Alcoholic and aqueous extracts contain high concentrations of flavonoids and phenolic acids. *S. officinalis* extracts demonstrate bactericidal and bacteriostatic activities against both Gram-positive and Gram-negative species. Antifungal and antiviral activities have also been reported (Al-Juraifani, 2009; Ghorbani and Esmaeilizadeh, 2017). Table 1 summarizes some of the main polyphenols found in Middle Eastern plants with antibacterial activity.

**Table 1:** A summary of some of the main polyphenols found in Middle Eastern plants with antibacterial activity.

**Plant name**	**Common name**	**Extract**	**Bacteria**	**MIC (mg/ml)**	**Zone of Inhibition (mm)**	**Compounds**	**Country**	**References**
*Eugenia caryophyllata*	Cloves	Aqueous 80°C	*B. subtilis*	1.6		Phenolics, flavonoids, terpenoids	Lebanon	Shehadi et al., 2014
		Aqueous 100°C		0.6				
*Mentha piperita*	Mint	Aqueous 80°C, 100°C	*B. subtilis*	0.5 × 10^2^				
		Aqueous 100°C		0.8 × 10^8^				
*Rosemarinus officinalis*	Rosemary	Aqueous: 80°C	*B. subtilis*	2.95		Phenolics, flavonoids, terpenoids	Lebanon	
		Aqueous 100°C		4.6				
		Ethanolic	*ESBL E. coli and K. pneumoniae*	0.0025–0.08				
			*B. subtilis, S. aureus, M. roseus*	0.05–0.2	8.0–29.0	Flavonoids, saponins, steroids, anthocyanins	Saudi Arabia	
			*E. coli, K. pneumoniae, S. dysenteriae, P. aeruginosa*		5.0–18.0			
*Prunus avium*	Cherry	Aqueous 80°C	*B. subtilis*	4		Phenolics	Lebanon	
		Aqueous 100°C		2.4				
*Solanum incanum* L.		Ethanolic	*S. aureus, E. coli, P. aeruginosa,Acinetobacter* sp., *K. pneumoniae, Proteus* sp., *Micrococcus* sp., *S. epidermidis, B. subtilis*	12.5	8.46–22.33	Phenolic acid, cinnamic acid, p-coumaric acid, ferulic acid, catechin, sinapic acid	Saudi Arabia	Alamri and Moustafa, 2012
*Ricinus communis* L.		Ethanolic		10	17.46–27.22			
*Allium ampeloprasum* var. pollum L.		Ethanolic		11.5	13.33–23			
*Syrian propolis*		Ethanolic	*S. aureus (MRSA)*		19–22	Phenolic acid, phenolic aldehydes, flavonoids, quinones	Syria	Harfouch et al., 2016
			*A. baumanii*		12.0–15.0			
			*E. coli*		13.0–17.0			
			*P. aeruginosa*		13			
*Matricaria aurea* L.	Golden chamomile	Methanolic	*B. subtilis*	0.4	24.83	Phenols, phenolic acids	Saudi Arabia	Rizwana et al., 2016
			*S. pyogenes*	0.4	23			
			*S. aureus*	50	21			
			*K. pneumoniae*	0.4	21			
			*Colletotrichium gleosporoides*	0.2–6.35	50–66.22			
*Punica granatum* L.	Pamogranate	Hydrochloric	*A. actinomycetemcomitans, P. intermedia, P. gingivalis*			Caffeic acid, gallic acid, epigallocatechin gallate	Middle East	Bhandari, 2012
			*Gram positive and Gram negative*	4.0–32.0			Jordan	Nimri et al., 1999
*Lawsonia inermis* Linn	Henna	Ethanolic	*P. aeruginosa*		24–26	5% 2-hydroxy-1,4-naphthaloquinone	Sultanate of Oman	Habbal et al., 2011
		Aqueous	*S. aureus*		7	Naphthaloquinone		Rathi et al., 2017
		Aqueous	*E. coli*		10			
			*B.vsubtilis, S. aureus, M. roseus*	0.05–0.2	8.0–29.0	Flavonoids, saponins, steroids, anthocyanins	Saudi Arabia	El Sayed and Aly, 2014
			*E. coli, K. pneumoniae, S. dysenteriae, P. aeruginosa*		5.0–18.0			
		Acetone	*B. subtilis*		36	Flavonoids, quinones, tannins, alkaloids	Yemen	Al Maqtari and Al. Maqtari, 2014
			*E. coli*		17			
			*L. monocytogenes*		30			
			*K. rhizophila*		34			
			*S. epidermidis*		24			
*Nigella sativa*	Habbah sauda, Kalonji, Fennel flower seeds, Onion seed, Nutmeg flower seed, Black cumin seed, black cumin, black caraway seed	Methanolic n-hexane	*S. aureus, S. mutans, S. mitis*		15–30 0–22	Thymoquinone, thymohydroquinones, dithymoquinone	Middle East	Sudhir et al., 2016
*Annona squamosa* L.			*S. aureus, E. faecalis, S. epidermidis, E. coli, P. aeruginosa*	50		Quinones, phenols, flavonoids	Lebanon	(Nasser et al., 2017)
*Premna resinosa*		Dichloromethane	*S. aureus, B. subtilis, E. coli, S. typhimurium, E. faecalis, S. flexneri, A. baumanii*	0.01–1			Saudi Arabia	Albadawi et al., 2017
*Pheonix dactylifera* L.	Date palm	Chloroform	imipenem resistant *P. aeruginosa*	0.05		Flavonoid glycosides: quercetin, apigenin, luteolin	Saudi Arabia	Selim et al., 2012
*Thymus vulgaris Boswelia carterii*	Thyme Myrrh	Ethanolic	*S. aureus, B. cereus, L. pneumophhila, Aspergillus flavus, Fusarium oxysporum*	2–4% (v/v)		Rosmarinic acid, caffeic acid, carnosol, flavonoids	Saudi Arabia	Al-Juraifani, 2009
*Plicosepalus acaciae*		Chloroform	*P. aeruginosa*		18	Alkaloids, Flavonoids, Tannins	Saudi Arabia	Saadabi et al., 2006
*Momordica balsamina*			*P. aeruginosa*		15			
*Cyperus rotrdus*			*E. coli*		15			
*Nymphea lotus*			*B. subtilis*		16			
*Vahila ddichotoma*			*S. aureus*		14			
*Olea europaea*	Olive	Methanolic	*S. aureus*	0.08	3.0–8.0	Flavonoids	Syria,Palestine	Malik, 2015
			*B. cereus*	0.04				
		Ethanolic	*ESBL E. coli and K. pneumoniae*	0.0025–0.08			Lebanon	Abdel-Massih et al., 2010; Daoud et al., 2011
			*Gram positive*		25mm	Tannins, Flavonoids, steroids, terpenoids, coumarins	Saudi Arabia	Khayat et al., 2018
			*S. aureu*	0.0312–0.0625				
			*E. coli*					
			*S. pyogenes*					
			*Salmonella* sp.					
			*P. aeruginosa*					
*Eruca sativa*	Arugula	Methanolic	*S. aureus*	0.06	3.0–8.0	Flavonoids	Syria, Palestine	Malik, 2015
			*B. cereus*	0.02				
*Azadirachta indica*	Neem		*B. subtilis, S. aureus, M. roseus*	0.05–0.2	8.0–29.0	Flavonoids, saponins, steroids, anthocyanins	Saudi Arabia	El Sayed and Aly, 2014
*Zingiber officinale Eucalptus globules*	Ginger		*E. coli, K. pneumoniae, S. dysenteriae, P. aeruginosa*		5.0–18.0			
*Conocarpus erectus* L.		Alcoholic	*S. cerevisiae*		14.3	Tannins	Saudi Arabia	Shohayeb et al., 2013
			*A. niger*		12.5			
			*P. chrysogenum*		13.3			
			*Gram positive (S. aureus, B. subtilis)*	0.21–1.33	21.0–23.0			
			*Gram negative*	0.42–8	11.0–18.0			
			*Acid fast (M. phlei)*	0.33–2.33	16.0–17.0			
*Curcuma Longa*	Turmeric	Aqueous	*S. pyogenes*		11	Tannins	Saudi Arabia	Al-Daihan et al., 2013
			*S. aureus*		11			
			*E. coli*		11			
			*P. aeruginosa*		14			
		Methanolic	*S. pyogenes*		19			
			*S. aureus*		15			
			*E. coli*		12			
			*P. aeruginosa*		12			
*Catha edulis*	Khat	Aqueous	*Streptocpccus sanguis TH-13, Streptococcus oralis SH-2, Fusobacterium nucleatum*					Al-Hebshi et al., 2006
Guandelia tournefortii L.								Darwish and Aburjai, 2010
*Pimpinella ansium* L.								
*Anagryis foetida*								
*Lepidium sativum*								
*Origanum syriacum* L.								
*Rosa damascene*								Abreu et al., 2012
*Thymbra spicata* L.		Petroleum ether	*MDR S. aureus*	6.25–12.5		Alkaloids	Middle east	Haroun and Al-Kayali, 2016
			*K. pneumoniae*	12.5				
*Salvadora persica* L.	Miswak	Aqueous	*S. aureus S. mutans S. faecalis S. pyogenis L. acidophilus P. aeruginosa C. albicans*	3.12 1.56 0.781 3.12 6.25 6.25 6.25	13.2 15.3 15.3 13.8 11.1 7.8 9.1	Saponins, alkaloids, volatile oils, terpenoids, flavonoids, carbohydrates	Middle East	Al-Bayati and Sulaiman, 2008AbdElRahman et al., 2002 Sher et al., 2011
		Ethanolic:stem	*A. actinomycetemcomitans*	50				
*Echium arabicum*		Methanolic	*Fusarium moniliforme*		23			Shahat et al., 2017
*Origanum majorana*		Ethanolic	*ESBL E. coli and K. pneumoniae*	0.0025–0.08			Lebanon	Abdel-Massih et al., 2010; Daoud et al., 2011
*Trigonella foenum-graecum*			*ESBL E. coli and K. pneumoniae*	0.0025–0.08			Lebanon	Abdel-Massih et al., 2010; Daoud et al., 2011
*Boswellia socotrana*		Mthanolic, aqueous	*Gram positive*	0.125- >1			Yemen	Mothana et al., 2009
*Quercus infectoria*	Aleppo oak		*Gram positive and Gram negative*	4.0–32.0			Jordan	Nimri et al., 1999
		Aqueous	*B. cerues*	0.00256	7.25		Syria, Iraq, Iran	Shariatifar et al., 2014
		Ethanolic		0.00512	16.85			
		Aqueous	*Y. enterocolitica*		14.45			
		Ethanolic			14.15			
*Rhus coriaria* L.	Sumac		*Gram positive and Gram negative*	4.0–32			Jordan	Kossah et al., 2013
			*Gram negative*	1–3.5			Syria	Al-Boushi et al., 2014
			*Gram positive*	0.5–1.5				
			*Yeast*	5.2–7				
*Thymus syriacus*			*Gram negative*	0.003125–0.0125		Thymol, carvacrol, dihydro-carvon, linalool	Syria	Al-Mariri et al., 2013

### Phenolics and Polyphenols

**Figure 1:**
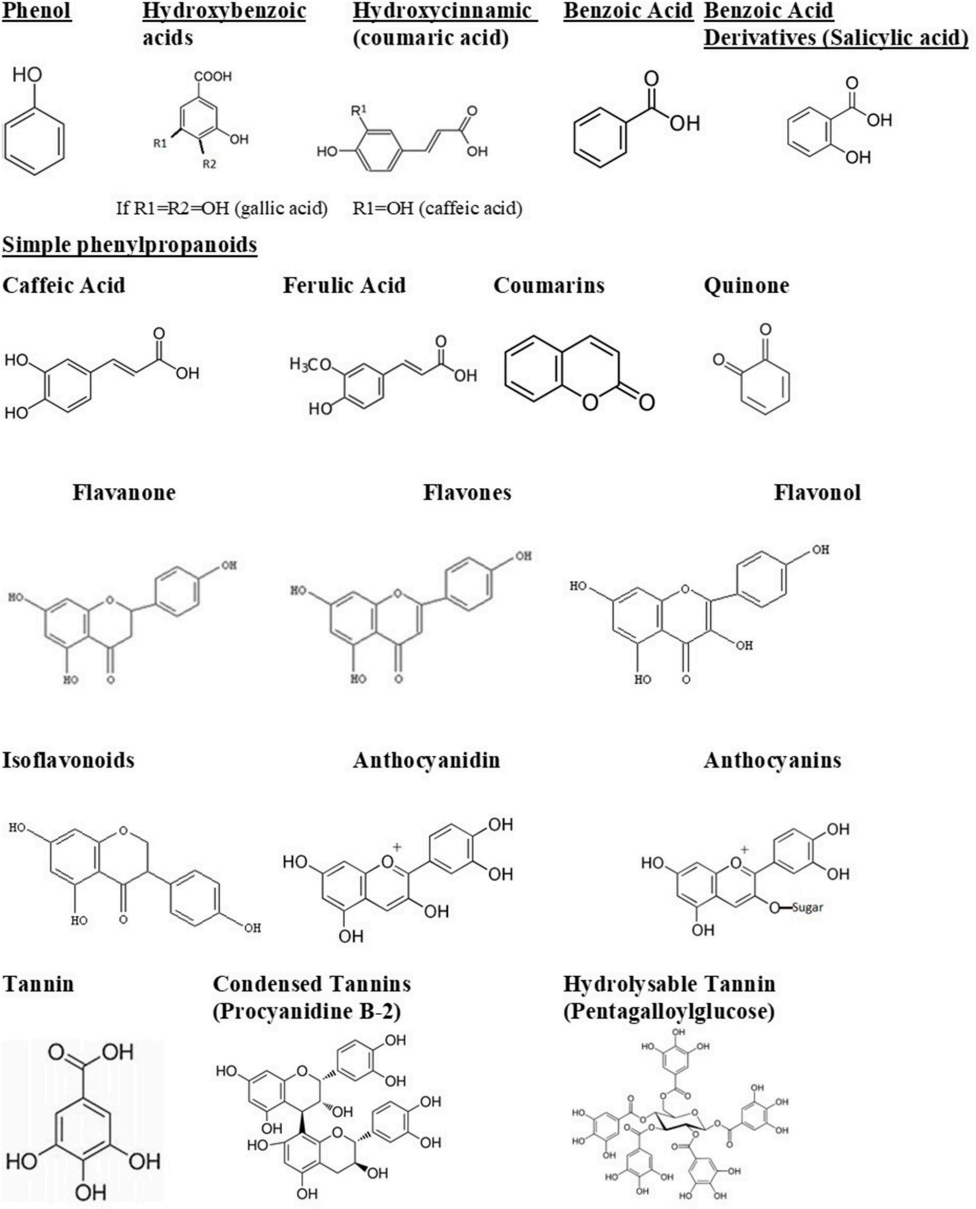
Phenolic compounds and derivatives found in medicinal plants (Taiz et al., 2015).

The bioavailability of polyphenols, or the amount of polyphenols that is absorbed unchanged, generally determines its biological activity. Polyphenols could also pass through the gastrointestinal system without being absorbed, thus affecting intestinal microbiota. This can lead to two consequences: first, polyphenols are modified into their active form; second, they change the composition of the intestinal microbiota, probably inhibiting pathogenic bacteria and enriching beneficial bacteria. Thus, polyphenols have a significant impact on the human host health (Abbas et al., 2017). They can be divided into several groups. The structures of main phenolic compounds and their derivatives are shown in Figure 1.

#### Simple Phenols and Phenolic Acids

Simple phenols and phenolic acids range from being a simple phenol ring with a single substitution like cinnamic and caffeic acids to having multiple substitutions and hydroxylations. There is evidence that the site and degree of hydroxylation correlates with the toxicity of the secondary metabolite. The metabolite seems to be more inhibitory the more oxidized the structure is (Cowan, 1999). Mechanisms of inhibition of phenolics include inhibiting enzymes. It is suggested that this inhibition takes place through reactions with sulfhydryl groups on the proteins (Cowan, 1999; Coppo and Marchese, 2014).

Four plants from Lebanese flora, cloves (*Eugenia caryophyllata*), mint (*Mentha piperita*), rosemary (*R. officinalis*), and cherry (*Prunus avium*) extracts were tested for their antimicrobial activity using the broth microdilution method against *B. subtilis*. Cloves had the highest antibacterial activity (MIC of 1.6 and 0.6 mg/ml after aqueous extractions at 80 and 100°C, respectively), followed by cherry (MIC of 4 and 2.4 mg/ml after extractions at 80 and 100°C, respectively). Phytochemical analysis demonstrated the presence of phenolics in cloves, cherry, and rosemary. There were also indication of flavonoids and terpenoids in cloves and rosemary (Shehadi et al., 2014).

Ethanolic extracts of three Saudi Arabian plants were tested for antimicrobial activity. The fruits of *Solanum incanum* L., leaves of *Ricinus communis* L., and *Allium ampeloprasum* var. porrum L. were tested against nine strains of bacteria. All three plants demonstrated antibacterial activity against *S. aureus, E. coli, P. aeruginosa, Acinetobacter* sp., *K. pneumoniae, Proteus* sp., *Micrococcus* sp., *Staphylococcus epidermidis*, and *B. subtilis*. The ethanolic leaf extract of *R. communis* exhibited the highest antibacterial activity with inhibition zones ranging between 17.46 and 27.22 mm at a concentration of 20 mg/ml and MIC of 10 mg/ml (Alamri and Moustafa, 2012). The ethanolic leaf extract of *A. ampeloprasum* var. porrum followed with a range of inhibition zones between 13.33 and 23 mm at a concentration of 23 mg/ml and MIC of 11.5 mg/ml. The highest activity was against *P. aeruginosa* and *Micrococcus* sp. Phytochemical screening using HPLC confirmed the presence of five phenolic compounds including phenolic acids cinnamic acid, p-coumaric acid, and ferulic acid as well as catechin and sinapic acid (Alamri and Moustafa, 2012).

Ethanolic extracts of *Syrian propolis* were able to inhibit growth of all *S. aureus* strains tested including MRSA strains. They were also able to inhibit *A. baumanii* with an inhibition zone of around 15 mm at 20% concentration. The ethanolic extract was less efficient on *P. aeruginosa* and *E. coli* strains. *Propolis* extracts contain several active compounds including phenolic acids and phenolic aldehydes as well as flavonoids and quinones (Harfouch et al., 2016).

*Matricaria aurea* L., native to Saudi Arabia, is a herb also known as golden chamomile. Antimicrobial screenings using several techniques including agar well-diffusion assay, tube dilution assay, and scanning electron microscopy revealed bigger inhibition zones in methanolic extracts than in ethanolic ones. *B. subtilis* was the most sensitive to the methanolic extracts with an inhibition zone of 24.83 mm, followed by *S. pyogenes* (23 mm), *S. aureus* (21 mm), and *K. pneumoniae* (21 mm; Rizwana et al., 2016). Methicillin-resistant *S. aureus* (MRSA) strains, *E. coli*, and *E. faecalis* demonstrated moderate sensitivity compared to the other strains tested. Antifungal activity was also assayed; *Colletotrichum gleosporoides* was the most sensitive with inhibition zones between 50 and 66.22 mm for different extracts. MICs with methanolic extract were 0.4 mg/ml for *B. subtilis, K. pneumoniae*, and *S. aureus* and a higher MIC of 50 mg/ml for MRSA strains. MICs for fungal strains were between 0.2 and 6.35 mg/ml for *Alternaria alternata* and *C. gleosporoides* and a higher MIC of 12.5–50 mg/ml for *A. niger* and *Aspergillus flavus*. Scanning electron microscope (SEM) imaging showed that the extract-treated cells demonstrated a change in shape and size as well as clustering. Cell damage and destruction was observed after treatment for 12 and 24 h. GC-MS analysis confirmed the presence of phenols and phenolic acids (Rizwana et al., 2016).

Pomegranate (*P. granatum* L). growing across the Middle East was tested for several medicinal uses including antimicrobial activity. Pomegranate juice is rich in polyphenols, especially caffeic acid, gallic acid, and epigallocatechin gallate (the main active component in green tea). Hydrochloric extracts of pomegranate decreased the colony-forming units/ml of dental plaque microorganisms by 84%; these microorganisms include *Aggregatibacter actinomycetemcomitans, P. intermedia*, and *P. gingivalis* (Bhandari, 2012).

#### Quinones

Quinones are characterized by having an aromatic ring with two ketone substitutions. Quinone antimicrobial activity comes from their ability to donate free radicals. They can also form irreversible complexes with amino acids in proteins, thus inactivating them. These properties of quinones make it possible for them to attack surface adhesions, polypeptides in the cell wall, and membrane enzymes. Quinones may also sequester substrates required by the microorganisms (Cowan, 1999).

Well-diffusion assay analysis of *Lawsonia inermis* Linn commonly known as henna demonstrated antibacterial activity against *P. aeruginosa*, with the highest activity shown by species from the Al-Sharqiya region (Sultanate of Oman). Henna leaves contain up to 5% of 2-hydroxy-1,4-naphthaloquinone by weight. The presence of quinone gives it its dyeing properties (Habbal et al., 2011; Rathi et al., 2017).

Several quinones present in *Nigella sativa* seeds from all over the Middle East proved to have antimicrobial effect against *S. aureus, S. mutans*, and *Streptococcus mitis*. Methanolic fractions had inhibition zones ranging between 15 and 30 mm, while n-hexane fractions ranged between 0 and 22 mm with variations between countries of origin. This antimicrobial activity is attributed to compounds like thymoquinone, thymohydroquinones, and dithymoquinone (Sudhir et al., 2016). Aqueous and methanolic extracts of the Lebanese *Annona squamosa* L. seeds are rich in quinones as well as phenols and flavonoids. The extracts demonstrate an MIC of 50 mg/ml against *S. aureus, E. faecalis, S. epidermidis, E. coli*, and *P. aeruginosa*. They also demonstrate an MBC of 100 mg/ml against the previously mentioned strains (Nasser et al., 2017).

#### Flavones, Flavonoids, and Flavonols

Flavones consist of an aromatic ring with only one ketone substitution. Hydroxylation yields a flavonol. Flavonoids occur as a C3–C6 unit linked to a phenolic ring that is also hydroxylated. These compounds are known to be synthesized in plants as a defense mechanism against microorganisms; thus, their antimicrobial effect is of no surprise. Their antimicrobial properties are probably because they form complexes with both extracellular and soluble proteins, as well as bacterial cell wall. They could also disrupt cell membranes if lipophilic enough (Cowan, 1999).

Phytochemical screening of the dichloromethane and ethyl acetate fractions of *Premna resinosa* grown in Saudi Arabia showed the presence of flavonoids. Agar diffusion assay demonstrated strong antimicrobial activity, with the highest activity in the dichloromethane fraction. MICs for the dichloromethane fraction ranged between 0.01 and 1 mg/mL against *S. aureus, B. subtilis, E. coli, S. typhimurium, E. faecalis, Shigella flexneri*, and *A. baumanii*. Gram-positive strains were more susceptible than Gram-negative strains (Albadawi et al., 2017).

The antimicrobial activity of *Phoenix dactylifera* L. (Date palm) growing in Al Madina, Saudi Arabia, was evaluated by analysis through MIC followed by scanning electron microscopy against imipenem-resistant *P. aeruginosa* (IRP). Active compounds were determined to be flavonoid glycosides, including quercetin, apigenin, and luteolin. The MIC of the choloroform fraction was 0.05 mg/ml and that of the MBC was 2 mg/ml. Biofilms produced by 12 IRP isolates were completely eradicated with 5% extract for 1 h. Analysis with SEM, after applying the flavonoid glycosides, demonstrated that *P. aeruginosa* cells started to deform at 30 min. At 60 min, the cells were completely deformed, thus suggesting that the mechanism of action is through forming pores in the cell wall and damaging it (Selim et al., 2012).

Thyme leaves (*Thymus vulgaris*) and myrrh exudates (*Boswellia carterii*), used in traditional medicine in Saudi Arabia, were tested against seven bacterial species including *S. aureus, B. cereus*, and *Legionella pneumophila* as well as two fungal species (*A. flavus* and *Fusarium oxysporum*). MICs ranged between 2 and 4% (v/v). Phytochemical screening demonstrated the presence of rosmarinic, caffeic, chlorgenic acid, carnosol, and flavonoids (Al-Juraifani, 2009).

Saadabi et al. (2006) tested 78 plant extracts from 26 plants growing in Saudi Arabia for antimicrobial activity. Alkaloids, flavonoids, and tannins were present in five of the most active plants including *Plicosepalus acaciae, Momordica balsamina, Cyperus rotrdus, Nymphea lotus*, and *Vahila dichotoma*. The chloroform extracts of *P. acaceiae* (against *P. aeruginosa*), *M. balsamina* (against *P. aeruginosa*), *C. rotrudus* (against *E. coli*), *N. lotus* (against *B. subtilis*), and *V. dichotoma* (against *S. aureus*) had inhibition zones of 18, 15, 15, 16, and 14 mm, respectively (Saadabi et al., 2006).

Olive (*O. europaea*) leaves and arugula (*Eruca sativa*) seeds native to Syria and Palestine were analyzed against *S. aureus* and *B. cereus*. Screening confirmed the presence of flavonoids in the extracts. Methanolic extracts demonstrated inhibition zone diameters of 3–8 mm. MICs for olive and arugula extracts were 80 and 60 μg/ml, respectively, against *S. aureus*, and 40 and 20 μg/ml against *B. cereus* (Malik, 2015).

Activities of *Azadirachta indica* (neem), *Zingiber officinale* (ginger), *Eucalyptus globulus, R. officinalis*, and *L. inermis* were analyzed for antimicrobial activity against Gram-positive bacteria (*B. subtilis, S. aureus*, and *M. roseus*) and Gram-negative bacteria (*E. coli, K. pneumoniae, Shigella dysenteriae*, and *P. aeruginosa*). Inhibition zones ranged between 8 and 29 mm against Gram-negative bacteria with the strongest activity exhibited by *A. indica* against *E. coli*. Inhibition zones against Gram-positive bacteria ranged between 5 and 18 mm; the strongest activity was exhibited by *R. officinalis* and *L. inermis* against *M. roseus* and by *E. globulus* against *S. aureus*. MICs ranged between 50 and 200 μg/ml with the strongest activity by *A. indica* against *E. coli, P. aeruginosa*, and *S. dysenteriae*. Phytochemical screening of the various plant extracts indicates the presence of flavonoids and tannins as well as saponins, steroids, and anthocyanins (El Sayed and Aly, 2014).

*Yemeni lawsoniainermis* L. leaves were extracted using several solvents including methanol, ethanol, acetone, and water. Phytochemical screening showed that all the leaves contained flavonoids as well as quinones, tannins, and alkaloids. Acetone extracts demonstrated the highest antibacterial activity with inhibition zones of 36, 17, 30, 34, and 24 mm against *B. subtilis, E. coli, Listeria monocytogenes, Kocuria rhizophila*, and *S. epidermidis*, respectively, at a concentration of 250 mg/ml. Methanolic extract demonstrated slightly weaker antibacterial activity followed by ethanolic extract. The aqueous extracts demonstrated very little or no antibacterial activity (Al Maqtari and Al. Maqtari, 2014).

#### Tannins

Tannins are a group of polymeric phenolics. They are divided into two main categories: hydrolyzable and condensed tannins. Gallic acid forms the basis of hydrolyzable tannins, usually esterified at multiple locations with D-glucose. Condensed tannins are more abundant and are derived from flavonoid monomers; they may be referred to as proanthocyanidins (Cowan, 1999).

Tannins' biological activity could be correlated to the patterns of oxidation and polymerization (Coppo and Marchese, 2014). Tannins exert their antimicrobial effect by complexing with proteins through both covalent and non-covalent interactions. They are also capable of complexing with polysaccharides. Evidence also exists for direct inactivation of microorganisms; it was shown that low concentrations of tannins changed the morphology of the germ tubes of *Crinipellis perniciosa*. In the case of condensed tannins, they have also been shown to be capable of binding cell walls of ruminal bacteria, inhibiting growth, and protease activity (Cowan, 1999).

*Olea* sp. growing in the Albaha region in Saudi Arabia demonstrated antimicrobial activity in both its aqueous and ethanolic extracts. Ethanolic extracts exhibited higher antimicrobial activity with an inhibitory zone of 25 mm against Gram-positive bacteria. MICs against *S. aureus, E. coli, S. pyogenes, Salmonella* sp., and *P. aeruginosa* ranged between 31.2 and 62.5 μg/ml. Phytochemical screening of *Olea* sp. demonstrated a high concentration of tannins as well as flavonoids, steroids, terpenoids, and coumarins (Khayat et al., 2018).

*Conocarpus erectus* L., a tropical and subtropical evergreen tree cultivated in Saudi Arabia, was evaluated using crude extracts from various parts of the plants as well as purified tannins. Tannins were active against three fungal species: *Saccharomyces cerevisiae, A. niger*, and *Penicillium chrysogenum* with inhibition zones of 14.3, 12.5, and 13.3 mm, respectively (Shohayeb et al., 2013). Alcoholic extracts of the flowers, fruit, leaf, and stem of the plant demonstrated activity only against *S. cerevisiae* with inhibition zones of 11.3, 13.3, 10.3, and 11.0 mm, respectively. When tested against bacteria, flowers and fruits of *C. erectus* were more active than other parts of the plant. Generally, Gram-positive bacteria including *S. aureus* and *B. subtilis* demonstrated higher sensitivity than Gram-negative bacteria. Gram-positive inhibition zones ranged between 21 and 23 mm and MICs ranged between 0.21 and 1.33 mg/ml. Inhibition zones of Gram-negative bacteria ranged between 11 and 18 mm and MICs between 0.42 and 8 mg/ml. Acid-fast bacteria *Mycobacterium phlei* had inhibition zones between 16 and 17 mm and MICs between 0.33 and 2.33 mg/ml (Shohayeb et al., 2013).

Methanolic and aqueous extracts of *Z. officinale* and *Curcuma longa* growing in Saudi Arabia exhibited antimicrobial activity. *Z. officinale* inhibited *S. pyogenes, S. aureus, E. coli*, and *P. aeruginosa* growth with inhibition zones of 10, 10, 9, and 14 mm, respectively, for the aqueous extract and 12, 12, 10, and 12 mm, respectively, for the methanolic extract. *C. longa* displayed inhibition zones of 11, 11, 11, and 14 mm for the aqueous extract and 19, 15, 12, and 12 mm for the methanolic extracts, respectively. Phytochemical screening indicated the presence of tannins in the extracts of the two plants (Al-Daihan et al., 2013).

The methanolic extracts of pomegranate (*P. granatum*) peel extracts contain high concentrations of hydrolyzable tannins, ellagic acid, and gallic acid. The extracts exhibited activity against *E. coli* O157:H7, *Salmonella* spp., *Vibrio cholerae* and *L. monocytogenes* (Coppo and Marchese, 2014).

#### Overview of Mechanism of Action of Polyphenols

Different mechanisms are thought to be responsible for antimicrobial activity of polyphenols. This includes enzyme inhibition by the oxidized compounds, possibly through reactions with proteins through SH- groups or through non-specific interactions (Mason and Wasserman, 1987). There are conflicting findings in literature about the degree of toxicity on microorganisms and the degree of hydroxylation. Some studies show that highly oxidized phenols (Scalbert, 1991) or those with more OHs are more inhibitory than those less oxidized are. Moreover, flavonoids with more (OH) groups had a greater antimicrobial activity (Sato et al., 1996). However, other studies show that flavonoids lacking hydroxyl groups on their β-rings were more active in membrane disruption in microbial targets (Chabot et al., 1992). Furthermore, some phenolics such as quinones act as a source of stable free radicals and bind irreversibly with proteins leading to its loss of function. Other targets are inactivating enzymes, binding to adhesins on the microbial cell surface, binding to cell wall proteins, and interacting with substrates, rendering them unavailable to the microorganism, complexing with metal ions, and others (Cowan, 1999).

### Alkaloids

#### Overview

**Figure 2:**
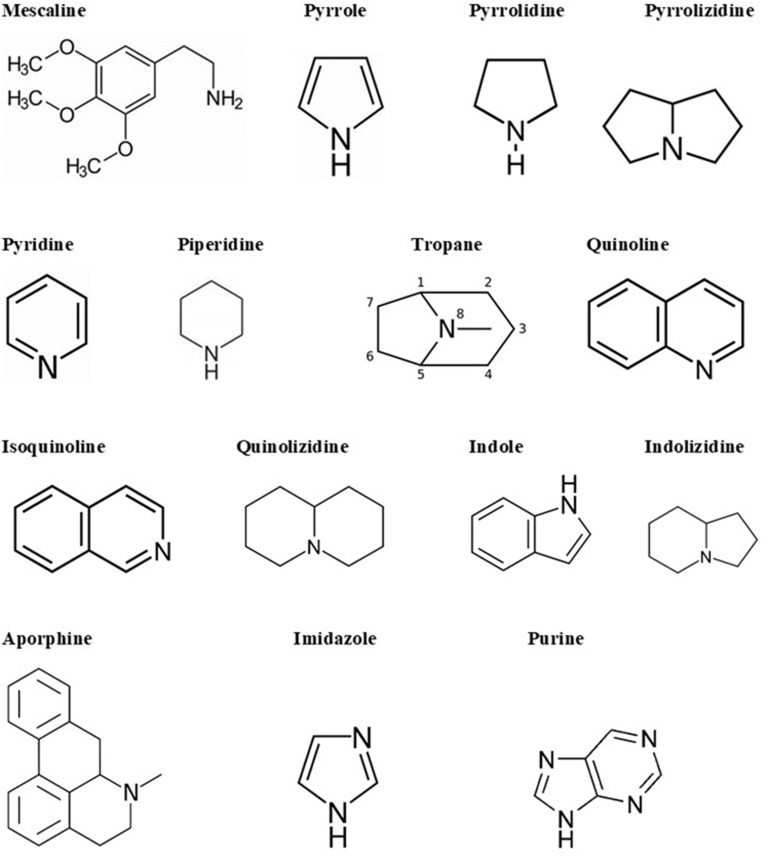
Alkaloid skeletal structures found in medicinal plants (Evans, 2009).

Alkaloids are a big and structurally diverse group of secondary metabolites that have microbial, plant, or animal origins. They can be found in around 300 plant families. However, some compounds are limited to specific families, such as hyoscyamine in *Solanaceae* (Cushnie et al., 2014). Though they are present in different parts of the plant, certain compounds are limited to a specific part, such as quinine in cinchona tree bark. Alkaloids are also found in terrestrial and in some marine animals. There are more than 18,000 alkaloids from different sources (Dembitsky, 2005). Alkaloids are heterocyclic structures containing one or more nitrogen atoms. They are classified based on their chemical structure or natural origin. Since some alkaloids are restricted to certain sources, classification due to their natural origin is feasible (Cushnie et al., 2014). There are two broad divisions in the classification according to the chemical structure. The first division contains the non-heterocyclic or atypical alkaloids, also called protoalkaloids or biological amines, such as hordenine or *N*-methyltyramine, colchicine, and erythromycin (an antibiotic; Evans, 2009). The second division includes the heterocyclic or typical alkaloids such as hygrines belonging to the pyrrole and pyrrolidine group, and quinine belonging to the quinoline group. The second division can be split into 14 groups based on the ring structure (Evans, 2009). The main structural units of alkaloids are shown in Figure 2.

There are several ways to name individual alkaloids. They can be named according to the organism from which they are isolated; for example, atropine is derived from *Atropa belladonna* (Cushnie et al., 2014). When several alkaloids are discovered from the same source, this requires the usage of a prefix or a more complex suffix, for example, quinine, hydroquinine, quinidine, or a series of letters, for instance, epicoccarine A, and epicoccarine B. Finally, alkaloids can be named based on their pharmacological activity (for example, emetine induces vomiting), the name of the source geographic location (for example, tasmanine was isolated from a Tasmanian plant), or after the name of their discoverer (for example, pelletierine after Prof. Pelletier; Cushnie et al., 2014).

Alkaloids are known in both traditional and modern medicine to have several pharmacological activities. Some alkaloids have been integrated into human culture as recreational drugs or abusive drugs such as caffeine, cocaine, and nicotine. Some alkaloids are known to be highly toxic, leading to several cases of human poisoning (Cushnie et al., 2014).

Alkaloids can form hydrogen bonds with enzymes, receptors, and proteins, since they have, in addition to functional groups, a proton accepting nitrogen atom, and one or even more proton donating amine hydrogen atoms. Alkaloids have many pharmacological properties, such as central nervous system stimulants (brucine), anticholinergic agents (atropine), oxytocic and vasoconstrictor activity (ergometrine), and antimalarial activity (quinine; Cushnie et al., 2014). Table 2 summarizes some of the main alkaloids found in Middle Eastern Plants with antibacterial activity.

**Table 2:** A summary of some of the main alkaloids found in Middle Eastern plants with antibacterial activity.

**Plant name**	**Common name**	**Extract**	**Species**	**MIC (mg/ml)**	**Disk difffusion (mm)**	**Compound**	**Country**	**Citation**
*Datura stramonium*	Jimson weed or Devil's Snare	Ethanolic	*E. col*		1.8	Daturine alkaloid, scopolamine alkaloid acids, tannins and fatty oil	Iraq	Altameme et al., 2015
			*P. aeruginosa*		1.4			
			*S. aureus*		1.3			
			*P. mirabilis*		2.01			
			*K. pneumonia*		1.7			
*Makhlaseh*		Aqueous and ethanolic		Aqueous fraction/Ethanolic fraction	Aqueous fraction/Ethanolic fra	Alkaloids, tannins, saponins, and phenolics	Iran	Behbahani and Fooladi, 2018
			*S. pyogenes*	8/4	13.60/14.30			
			*S. epidermidis*	8/4	13.40/14.30			
			*S. aureus*	16/8	13.20/13.70			
			*E. aerogenes*	32/16	9.20/11.90			
			*E. coli*	32/16	10.00/11.60			
			*S. flexneri*	16/16	11.30/12.70			
*Peganum harmala*	Wild rue, Syrian rue, African rue, Esfand or Harmel	Methanolic	*MRSAB. anthracisE. coli S. typhi*	0.625/0.625 2.5/1.25 0.625/0.625 0.625/0.625		Several alkaloids	Middle East, Mediterranean, Pakistan, India, Iran, North Africa, introduced to Australia and America	Darabpour et al., 2011
*Peganum harmala*	Wild rue, Syrian rue, African rue, Esfand or Harmel	Mixtures of harmala alkaloids fractionated from the methanolic extract		Individual and binary mixtures of harmala alkaloids	Mixtures of harmala alkaloids	Around 2 to 6% of alkaloids are found in the seed of P. harmala, they are mostly β-carbolines such as harmaline, harman, harmalol, and harmine	Middle East, Mediterranean, Pakistan, India, Iran, North Africa, introduced to Australia and America	Nenaah, 2010
			*E. coli*	0.333–1.5	Ranging from 10.5 to 31.5			
			*P. vulgaris*					
			*S. aureus*					
			*B. subitilis*					
			*A. niger*					
			*C. albicans*					
*Phoenix dactylifera L*.	Date Palm	Water, methanolic, acetone			Leaves (water, methanolic, acetone)/Pits (water, methanolic, acetone)	Alkaloids, flavonoids, tannins, steroids, carbohydrates, and vitamins	Majority of Arabian Peninsula	Perveen et al., 2012
			*E. coli*		(5.7, 17, 16.3)/(7, 20.7, 18.3)			
			*S. aureus*		(5.7, 15.7, 17.7)/(6, 21, 20)			
			*B. subtilis*		(8.7, 20.7, 19.7)/(6.7, 23.7, 23.3)			
			*S. pyogenes*		(7, 20.3, 20)/(7, 29.3, 29.3)			
			*P. aeruginosa*		(nd, 19.7, 18.7)/(nd, 19.7, 14.3)			
			*E. faecalis*		(nd,nd,nd)/(nd,nd,nd)			
			*S. flexeneri*		(9.3, 17.7, 21)/(6, 19.3, 17)			
*Achillea Millefolium L*.	Yarrow	Alcoholic and aqueous	*S. enterica enterica (typhimurium)*		Alcoholic/Aqueous	Aerial parts are known to have volatile oil rich in alkaloids and sesquiterpene lactones	Asia, North America, and Europe	Hasson, 2011
					9/12			
			*S. flexneri*		8/10			
			*P. aeruginosa*		30/12			
			*S. aureus*		24/nd			
			*E. faecalis*		8/nd			
			*M. luteus*		23/10			
*Tribulus terrestris L*.	Puncture Vine, Caltrop, Yellow Vine, Goathead	Aqueous, ethanolic, and chloroform		Fruits (aqueous, ethanolic, chloroform)/leaves (aqueous, ethanolic, chloroform)/roots (aqueous, ethanolic, chloroform)		Mixture of beta-carboline alkaloids, flavonoid glycosides, furostanol, phytosterols, spirostanol saponins, and some amides	Warm regions of Asia, Australia, Africa, America, and Europe	Al-Bayati and Al-Mola, 2008
			*S. aureus*	(2.5, 0.62, 0.62)/(2.5, 1.25, 2.5)/(>5, 0.62, 0.31)				
			*B. subtilis*	(1.25, 0.15, 0.31)/(1.25, 0.31, 0.31)/(2.5, 0.62, 0.31)				
			*B. cereus*	(1.25, 0.15, 0.31)/(2.5, 0.62, 0.62)/(5, 1.25, 0.62)				
			*C. diphtheriae*	(0.62, 0.15, 0.31)/(1.25, 0.62, 1.25)/(2.5, 1.25, 0.62)				
			*P. vulgaris*	(>5, 0.15, 1.25)/(2.5, 0.31, 1.25)/(>5, 2.5, 1.25)				
			*E. coli*	(1.25, 0.62, 0.62)/(2.5, 0.62, 1.25)/(>5, 2.5, 1.25)				
			*S. marcescens*	(>5, 2.5, 2.5)/(>5, 5, >5)/(>5, >5, >5)				
			*S. typhimurium*	(5, 1.25, 2.5)/(>5, 1.25, 5)/(>5, >5, 2.5)				
			*K. pneumoniae*	(2.5, 1.25, 1.25)/(2.5, 0.31, 2.5)/(>5, 2.5, 1.25)				
			*P. aeruginosa*	(>5, 1.25, 2.5)/(>5, 1.25, 2.5)/(5, 2.5, 1.25)				
			*C. albicans*	(>5, 0.62, 2.5)/(>5, 1.25, 2.5)/(>5, 5, 2.5)				
*Tribulus terrestris L*.	Puncture Vine, Caltrop, Yellow Vine and Goathead	Methanolic		Methanolic (fruits/stems plus leaves/roots)	Methanolic (fruits/stems plus leaves/roots)	Alkaloids, saponins, amides, and diosgenins	Warm regions of Asia, Australia, Africa, America, and Europe	Kianbakht and Jahaniani, 2003
			*S. aureus*	2/2/4	27.8/28.2/26.8			
			*E. faecalis*	2/2/4	14.6/15.7/14.3			
			*E. coli*	2/2/4	25.7/22.3/20.9			
			*P. aeruginosa*	2/2/2	26.2/23.8/21.3			
*Datura innoxia*	Thorn apple	Methanolic			Methanolic (2.5, 1.25, 0.75 mg/ml)	Alkaloids and steroids	Iran	Eftekhar et al., 2005
			*B. subti*		18, 14, 10			
			*S. aureus*		12, 8, 0			
			*E. coli*		9, 0, 0			
			*E. faecalis*		13, 10, 7			
			*P. aeruginosa*		0, 0, 0			
*Datura stramonium*	Jimson weed	Methanolic			Methanolic (2.5, 1.25, 0.75 mg/ml)	Alkaloids and steroids	Iran	
			*B. subti*		11, 8, 7			
			*S. aureus*		10, 7, 0			
			*E. coli*		0, 0, 0			
			*E. faecalis*		9, 0, 0			
			*P. aeruginosa*		0, 0, 0			
*Catharanthus roseus*	Rose periwinkle	Ethanolic	*E. coli*		11	Contains more than 130 different alkaloids	Saudi Arabia	Khalil, 2012
			*S. aureus*		15			
			*C. albicans*		12			
*Bunium persicum*	Black zira	Black Zira essential oil	*E. aerogenes*	8	7.1	Its essential oils contain alkaloids, phenolics, saponins, tannins, and flavonoids	Iran	Noshad et al., 2018
			*P. aeruginosa*	4	7.2			
			*S. flexneri*	2	7.9			
			*E. coli*	2	8.8			
			*S. epidermidis*	1	10			
			*S. pyogenes*	1	9.3			
			*C. albicans*	1	10.1			
*Solanum nigrum*	Black nightshade	Alkaloid leaf methanolic	*K. pneumonia*		1.8	Twenty three chemical alkaloids	Iraq	Jasim et al., 2015
			*P. aeruginosa*		1.4			
			*S. aureus*		1.9			
			*P. mirabilis*		2.01			
			*E. coli*		1.7			
*Nigella sativa L*.	Black cumin	Essential oils	*S. mitis*	4.25	11.5	15 amino acids, proteins, carbohydrates, both fixed oils (84% fatty acids, including linolenic and oleic) and volatile oils, alkaloids, saponins, crude fiber, as well as minerals, such as calcium, iron, sodium and potassium	Tunisia	Harzallah et al., 2011
			*S. mitis*	2.13	15.5			
			*S. mitis*	2.13	14.5			
			*S. mitis*	4.25	11.5			
			*S. mitis*	2.13	14.5			
			*S. mutans*	2.13	14.5			
			*S. mutans*	2.13	15.5			
			*S. mutans*	2.13	15			
			*S. mutans*	2.13	14			
			*S. constellatus*	2.13	14.5			
			*S. constellatus*	4.25	15.5			
			*S. constellatus*	4.25	11.5			
			*S. constellatus*	4.25	14.5			
			*S. oralis*	2.13	13.5			
			*S. oralis*	4.25	15.5			
			*S. oralis*	4.25	14.5			
			*S. anginosus*	4.25	11.5			
			*S. anginosus*	8.5	12.5			
			*S. anginosus*	4.25	12.5			
			*E. faecalis*	8.5	0			
			*E. faecalis*	>8.5	0			
			*E. faecalis*	>8.5	0			
			*E. faecalis*	>8.5	0			
			*S. salivarius*	4.25	0			
			*S. salivarius*	4.25	0			
			*G. haemolysans*	2.13	10.5			
			*G. haemolysans*	4.25	15.5			
			*G. morbillorum*	2.13	14.5			
			*S. sanguis*	8.5	10.5			
			*S. sanguis*	8.5	11.5			
*N. sativa L*.	Black cumin	Alkaloid	*B. subtilis*	0.064		Contains alkaloids, carbohydrates, saponins, proteins, minerals, and volatile oils in its seeds	Middle East, Asia, Eastern Europe, and Africa	Shohayeb and Halawani, 2012
			*S. aureus*	0.064				
			*M. phlei*	4				
			*P. aeruginosa*	2				
			*E. coli*	8				
			*K. pneumoniae*	8				
			*S. flexneri*	4				
			*S. typhimurium*	4				
*Juniperus excels*	Greek juniper	Hexane, chloroform, ethyl acetate, hydroalcoholic			Hexane/Chloroform/Ethyl acetate/Hydroalcoholic	Alkaloids, flavonoids, saponins, steroids, triterpenoids, glycosides, and tannins	Turkey, Lebanon, Syria, and other Balkan countries	Weli et al., 2014
			*E. coli*		nd/8/7/nd			
			*S. aureus*		13/8/11/9			
			*P. aeruginosa*		8/10/10/7			
*Prosopis farcta*	Syrian mesquite	Aqueous	*E. coli*	0.0015	8	Alkaloids, glycosides, and tannins	Iraq	Al-Ameri, 2006
			*P. aeruginosa*	0.0015	9			
			*S. aureus*	0.0015	9.3			
			*S. pyogenes*	0.025	6.6			
			*C. albicans*	0.0015	11.6			
			*C. cladosporioides*	0.0062	7			
			*C. neoformans*	0.0007	9			
			*T. mentagrophytes*	0.0062	8.3			
			*T. violaceum*	0.0007	8.3			
*Conocarpus lancifolius Engl*.		Alkaloid	*B. subtilis*	0.02	16	Alkaloid	Yemen, Somalia, and Djibouti	Ali et al., 2013
			*B. cereus*	0.02	13			
			*S. aureus*	0.05	12			
			*P. aeruginosa*	0.05	14			
			*S. marcescens*	0.05	15			
			*E. coli*	0.01	16			
			*A. tumefaciens*	0.02	17			
			*E. amylovora*	> 0.2	nd			
*Oliveria decumbens*	Mooshkorok	Essential oil	*P. aeruginosa (clinical strain)*	8	7	Its essential oils contain alkaloids, flavones, saponins, and phenolics	Grows in the warm area of west and south Iran	Behbahani et al., 2018
			*E. coli (clinical strain)*	4	9.2			
			*S. epidermidis (clinical strain)*	2	10.8			
			*S. pyogenes (clinical strain)*	1	12.1			
			*P. aeruginosa (standard strain)*	4	8.6			
			*E. coli (standard strain)*	4	9.9			
			*S. epidermidis (standard strain)*	1	11.9			
			*S. pyogenes (standard strain)*	1	13.2			
*Ficus sycomorus*	Fig mulberry	Hexane, chloroform, ethyl acetate, butanolic, methanolic, water			Hexane, Chloroform, Ethyl acetate, Butanol, Methanolic, W	Alkaloids, phenolic compounds, flavonoids, and tannins	Oman	Al-Matani et al., 2015
			*E. coli*		9/10/7/7/7/7			
			*Proteus* spp.		7/7/7/6/7/7			
			*S. aureus*		9/6/8/7/8/10			
			*H. influenzae*		8/6/nd/nd/nd/5			
*Papaver rhoeas L*.	Corn rose	Methanolic and alkaloid		Methanol (Plant 1 to Plant11)/Alkaloid (Plant 1 to Plant11)		Alkaloids	Turkey and and Cyprus	Çoban et al., 2017
			*S. aureus*	(nd to 1.25)/(nd to 0.312)				
			*S. epidermidis*	(nd to 0.625)/(nd to 0.625)				
			*K. pneumoniae*	(nd to 0.312)/(nd to 0.039)				
			*P. mirabilis*	(nd)/(nd)				
			*E. coli*	(nd)/(nd to 0.312)				
			*P. aeruginosa*	(nd to 0.312)/(nd to 0.156)				
			*C. albicans*	(nd to 0.312)/(nd to 0.625)				
*Amaryllis belladonna L*.	Jersey lily	Alkaloids		(-)-Lycorine/(-)-Amarbellisine/(-)-Hippeastrine/(-)-Pancracine/(+)-Vittatine/(+)-11-Hydroxyvittatine	(-)-Lycorine/(-)-Amarbellisine/(-)-Hippeastrine/(-)-Pancracine/(+)-Vittatine/(+)-11-Hydroxyvittatin	Exclusive source of Amaryllidaceae alkaloids	Egypt	Evidente et al., 2004
			*S. aureus*	nd/0.125/0.125/0.188/0.063/0.219	nd/22/nd/22/19/17			
			*E. coli*		nd/22/nd/nd/nd/22/nd			
			*P. aeruginosa*		nd/nd/nd/ 16/nd/ nd			
			*C. albicans*	0.039/0.063/0.125/0.188/0.031/0.156	40/24/25/15/17/20			
*Zingiber officinale*	Ginger	Aqueous and methanolic			Aqueous/Methanolic	Alkaloids, carbohydrates and saponins	Saudi Arabia	Al-Daihan et al., 2013
			*S. pyogenes*		10/12			
			*S. aureus*		10/12			
			*E. coli*		9/10			
			*P. aeruginosa*		14/12			
*Commiphora molmol*	Myrrh gum	Aqueous and methanolic			Aqueous/Methanolic	Alkaloids, carbohydrates and saponins	Saudi Arabia	
			*S. pyogenes*		12/8.5			
			*S. aureus*		14/19			
			*E. coli*		9/9			
			*P. aeruginosa*		12/13			
*Polygonum aviculare L*	Knotgrass	Water, acetone, chloroform, and ethanolic		Stem chloroform fraction/Leaf chloroform fraction	Stem (Water, Acetone, Chloroform, Ethanolic) and Leaf (Water, Acetone, Chloroform, Ethanolic)	Alkaloids, tannins, flavonoids, saponins, and sesquiterpenes	Egypt	Salama and Marraiki, 2010
			*E. coli*	15/18	(22, 18, 27, 23)/(9, 7, 11, 7)			
			*P. mirabilis*	15/20	(24, 15, 28, 23)/(8, 5, 8, 4)			
			*P. aeruginosa*	15/18	(21, 19, 23, 22)/(7, 5, 9, 5)			
			*S. typhi*	10/15	(22, 14, 24, 23)/(8, 5, 11, 7)			
			*S. paratyphi*	8/15	(23, 15, 23, 20)/(5, 7, 12, 8)			
			*S. flexneri*	8/10	(19, 16, 21, 16)/(6, 4, 11, 6)			
			*S. aureus*	18/20	(22, 17, 24, 21)/(7, 2, 10, 8)			
			*B. subtilis*	8/18	(25, 20, 25, 22)/(5, 5, 8, 7)			
			*S. pyogenes*	10/15	(18, 15, 23, 20)/(4, 8, 12, 9)			
			*A. flavus*	5/8	(14, 10, 17, 13)/(6, 6, 10, 9)			
			*A. fumigatus*	1/5	(15, 8, 18, 14)/(3, 4, 9, 8)			
			*A. niger*	1/5	(13, 7, 14, 15)/(2, 5, 8, 6)			
			*C. albicans*	0/0	(0, 0, 0, 0)/(0, 0, 0, 0)			
*Chamomilla recutita*	Chamomile	Methanolic	*B. subtilis*		nd	Alkaloids, triterpenes, phenolic compounds, carbohydrates, and saponins	Egypt	Abdel-Hameed et al., 2008
			*E. coli*		14			
			*S. aureus*		nd			
			*C. albicans*		14			
			*A. niger*		nd			
*Buddleja hybrida*	Summer lilac	Methanolic	*B. subtilis*		14		Egypt	
			*E. coli*		16			
			*S. aureus*		16			
			*C. albicans*		nd			
			*A. niger*		nd			
*Glinus lotoides*	Damascisa	Methanolic	*B. subtilis*		8		Egypt	
			*E. coli*		12			
			*S. aureus*		8			
			*C. albicans*		10			
			*A. niger*		18			

#### Antibacterial Activity of Alkaloids Derived From Middle Eastern Plants

The antibacterial activity of the alkaloids found in the ethanolic extract of *Datura stramonium*, an annual herb commonly found in Baghdad district, was tested using the agar well-diffusion method against *E. coli, P. aeruginosa, S. aureus, Proteus mirabilis*, and *K. pneumoniae*. The tested microorganisms were more sensitive to the ethanolic leaf extract as compared to standard antibiotics (Altameme et al., 2015). Daturine alkaloid, scopolamine alkaloid acids, tannins, and fatty oil are known to be found in the leaves, seeds, and roots of this plant. GC-MS of the ethanolic extract of *D. stramonium* showed the presence of eight alkaloid compounds (Altameme et al., 2015).

GC-MS analysis of the aqueous (evaporated using a rotary evaporator and reconstituted in an appropriate organic solvent) and ethanolic extract of the Iranian herbaceous plant Makhlaseh, member of Asteraceae, revealed the presence of alkaloids, tannins, saponins, and phenolics (Behbahani and Fooladi, 2018). Alkaloids and phenolics were at higher concentration in the ethanolic extract. The pour plate method showed that both the aqueous and ethanolic Makhlaseh extracts (2 mg/ml) reduced the growth of *S. epidermidis* and *S. pyogenes*; whereas no antibacterial effect was observed against the Gram-negative bacteria tested. Similarly, the disk diffusion method showed that the aqueous and the ethanolic extracts had a great effect at 40 mg/ml against Gram-positive bacteria tested but not against Gram-negative bacteria. The MIC results for the aqueous extract of Makhlaseh for *S. pyogenes, S. epidermidis, S. aureus, S. flexneri, E. coli*, and *Enterobacter aerogenes* were 8, 8, 16, 16, 32, and 32 mg/ml, respectively, and those of the ethanolic extract were 4, 4, 8, 16, 16, and 16 mg/ml, respectively (Behbahani and Fooladi, 2018).

*Peganum harmala* is found in the Middle East, Mediterranean, Pakistan, India, Iran, and North Africa and has been introduced to Australia and America (Darabpour et al., 2011). Roots and seeds of *P. harmala* are rich in alkaloids. A broad antibacterial activity was observed for the methanolic extracts from its roots and seeds against 13 MDR Gram-positive (*S. pyogenes, S. epidermidis, S. aureus, Bacillus pumilus, Bacillus anthracis, B. cereus*, and *L. monocytogenes*) and Gram-negative (*K. pneumoniae, P. aeruginosa, Salmonella typhi, Brucella melitensis, P. mirabilis*, and *E. coli*) bacterial clinical isolates. The stem, flower, and leaf extracts of *P. harmala* showed a poor antibacterial activity using the disk diffusion method. The lowest MIC and MBC of both root and seed extracts of *P. harmala* were against MRSA (0.625 mg/ml). The seed extract gave a similar MIC with *E. coli* and *S. typhi* (0.625 mg/ml; Darabpour et al., 2011). Around 2–6% of alkaloids are found in the seed of *P. harmala*; they are mostly β-carbolines such as harmaline, harman, harmalol, and harmine (Nenaah, 2010). The isolated β-carboline alkaloids had an antimicrobial effect against *E. coli, P. vulgaris, S. aureus, B. subtilis, A. niger*, and *C. albicans* with inhibition zone diameters ranging from 10.5 to 31.5 mm using the agar diffusion method. The MIC results of the alkaloids ranged between 0.333 and 1.5 mg/ml (Nenaah, 2010). Synergy tests showed that a combination of the root and seed extracts with novobiocin against MRSA, *E. coli, K. pneumoniae*, and *B. anthracis* showed a good synergistic effect (Darabpour et al., 2011). Moreover, the combination of colistin with these two extracts against the colistin-resistant *E. coli* and *L. monocytogenes* strains showed an excellent antibacterial activity (Darabpour et al., 2011). Furthermore, the combination of carbenicillin with the root extract showed a better antibacterial activity against MRSA and *B. anthracis* compared to that with the seed extract (Darabpour et al., 2011).

Date palm is abundant in the majority of Arabian Peninsula and it is known to be as one of the most important commercial crops. Date palms contain alkaloids, flavonoids, tannins, steroids, carbohydrates, and vitamins (Perveen et al., 2012). The antibacterial activity of the acetone, methanol, and aqueous extracts of three varieties of *P. dactylifera* L. (date palm) against ATCC strains of *E. coli, P. aeruginosa, S. aureus, B. subtilis*, and *E. faecalis* and clinical isolates of *S. flexneri* and *S. pyogenes* was tested using the agar well-diffusion method (Perveen et al., 2012). The methanol and acetone extracts showed a good antibacterial activity against all the tested strains, except for *E. faecalis* (Perveen et al., 2012). The pit extract had a better antibacterial activity compared to the leaf extract. MICs for the most sensitive strain tested, *S. pyogenes*, were 1.4, 1.6, 1.15, and 1.33 mg/ml for the acetone pit extract, acetone leaf extract, methanol pit extract, and methanol leaf extracts, respectively (Perveen et al., 2012).

The antibacterial activity of the aqueous and alcoholic extracts of *Achillea millefolium* L. (Yarrow) against *P. aeruginosa, S. flexneri, S. aureus, Salmonella enterica* subsp. *enterica* (Typhimurium), *M. luteus*, and *E. faecalis* was tested (Hasson, 2011). Yarrows grow in Asia, North America, and Europe, and its aerial parts are known to have volatile oil rich in alkaloids and sesquiterpene lactones (Hasson, 2011). The Kirby Bauer disk diffusion test results showed a broad spectrum of antibacterial activity for the aqueous and alcoholic flower extract of yarrow against the tested bacteria at different rates (Hasson, 2011). The best inhibitory effect of the alcoholic extract was against *P. aeruginosa, S. aureus*, and *M. luteus*, whereas the aqueous extract showed a slight inhibition against *P. aeruginosa* and *M. luteus* (Hasson, 2011).

*Tribulus terrestris* L. is found in warm regions of Asia, Australia, Africa, America, and Europe (Kianbakht and Jahaniani, 2003). It is used as a urinary anti-infective in folk medicine in Iraq. It contains alkaloids, saponins, amides, and diosgenins (Kianbakht and Jahaniani, 2003). *T. terrestris* fruits, root, and leaves contain a mixture of beta-carboline alkaloids, flavonoid glycosides, furostanol, phytosterols, spirostanol saponins, and some amides (Al-Bayati and Al-Mola, 2008). The aqueous, chloroform, and ethanolic extract of *T*. *terrestris* fruits had a good antibacterial activity against *S. aureus, Corynebacterium diphtheriae, B. subtilis, B. cereus, E. coli, K. pneumoniae, P. vulgaris*, and *Salmonella typhimurium* with the best MIC as 0.62, 0.31, and 0.15 mg/ml, respectively (Al-Bayati and Al-Mola, 2008). The aqueous extract of *T*. *terrestris* leaves showed similar results to the fruit extracts; however, the chloroform and ethanolic leaf extracts had lower activity (Al-Bayati and Al-Mola, 2008). A moderate to no activity was exhibited by the three extracts from *T*. *terrestris* roots against the tested strains (Al-Bayati and Al-Mola, 2008). The best antimicrobial activity was exhibited by the fruit's ethanol extract with MIC of 0.15 mg/ml against *B. subtilis, B. cereus, P. vulgaris, C. diphtheria*, and *C. albicans*. Similarly, the methanolic extracts from all the parts of the Turkish and Iranian *T. terrestris* had a considerable antibacterial activity with inhibition zone diameters ranging between 14.3 and 28.2 mm (Kianbakht and Jahaniani, 2003).

The antibacterial activity of the methanolic extract of *Datura innoxia* and *D. stramonium*, found in Iran, was tested using the paper disk diffusion method (Eftekhar et al., 2005). These plants are rich in alkaloids and steroids. *D. innoxia* extract exhibited a dose-dependent antibacterial activity, against *S. aureus, E. faecalis*, and *B. subtilis*, with optimum activity at 2.5 mg/ml (Eftekhar et al., 2005). The methanolic extract of *D. stramonium* showed a slight antibacterial activity at this concentration. The methanolic extracts of both plants showed a small or negligible activity against *P. aeruginosa* and *E. coli* (Eftekhar et al., 2005).

The antimicrobial activity of the ethanolic leaf extract *Catharanthus roseus* (100 mg/ml) was tested. *C. roseus* contains more than 130 different alkaloids and is found in Saudi Arabia. Using the disk diffusion method, the inhibition zone was 15, 12, and 11 mm against *S. aureus, C. albicans*, and *E. coli*, respectively (Khalil, 2012).

Black Zira, *Bunium persicum*, grows in Iran, and its essential oils contain alkaloids, phenolics, saponins, tannins, and flavonoids (Noshad et al., 2018). The disk diffusion agar results reveal that the Black Zira's essential oil has a good antimicrobial activity against *E. coli, S. pyogenes, S. epidermidis*, and *C. albicans*. The MIC results ranged from 1 to 8 mg/ml, while the MBC values ranged from 1 to 16 mg/ml (Noshad et al., 2018).

The antibacterial activity of alkaloid extracts from the methanolic extract of leaves of *Solanum nigrum*, found in Iraq, was tested against *K. pneumonia, P. aeruginosa, S. aureus, P. mirabilis*, and *E. coli* (Jasim et al., 2015). The well-diffusion method results showed that all the strains were sensitive to the methanolic leaf extract and the activity was better than that of tested antibiotics (kanamycin, cefotaxime, penicillin, streptomycin, and rifampin; Jasim et al., 2015).

*N. sativa* has been used for centuries in the Middle East, Asia, Eastern Europe, and Africa as a natural remedy against many diseases (Shohayeb and Halawani, 2012). *N. sativa* contains alkaloids, carbohydrates, saponins, proteins, minerals, and volatile oils in its seeds (Harzallah et al., 2011). The antibacterial activity of *N. sativa* L. (Black cumin) essential oils and thymoquinone against 30 human cariogenic strains was studied using the agar disk diffusion assay and MIC. Black cumin essential oils (2.43 mg/disc), found in Tunisia, were very effective against *S. mitis, S. mutans, Streptococcus oralis, Streptococcus constellatus*, and *Gemella haemolysans* (inhibition zone 13.5 to 15.5 mm; Harzallah et al., 2011). The best antimicrobial activity of the essential oils was against *G. haemolysans, S. mutans, S. constellatus*, and *S. mitis* with an MIC value of 2.13 mg/ml (Harzallah et al., 2011). Thymoquinone was effective against all the tested strains, and its highest antibacterial activity was against *S. constellatus* with an MIC value of 4 μg/ml, where it gave results comparable to those of erythromycin (Harzallah et al., 2011). The MIC results of the alkaloid extract of *N. sativa* against *B. subtilis, S. aureus, M. phlei, P. aeruginosa, E. coli, K. pneumoniae, S. flexneri*, and *S. typhimurium* were 64, 64, 4,000, 2,000, 8,000, 8,000, 4,000, and 4,000 μg/ml, respectively (Shohayeb and Halawani, 2012).

*J. excelsa* is found in Turkey, Lebanon, Syria, and other Balkan countries and is rich in alkaloids, flavonoids, saponins, steroids, triterpenoids, glycosides, and tannins (Weli et al., 2014). Using the disk diffusion method, the chloroform and ethyl acetate extracts had inhibition diameters ranging between 6 and 13 mm for *E. coli, S. aureus*, and *P. aeruginosa*. Similarly, the hexane and hydroalcoholic extracts had the same inhibition diameters except on *E. coli*, where no activity was observed (Weli et al., 2014).

*Prosopis farcta*, found in Iraq, is rich in alkaloids, glycosides, and tannins (Al-Ameri, 2006). The MIC results of the aqueous extract of *P. farcta* are 1.5 μg/ml for *E. coli, P. aeruginosa*, and *S. aureus* and 25 μg/ml for *S. pyogenes* (Al-Ameri, 2006). Moreover, the fungal strains exhibited an MIC of 0.7 μg/ml for *Cryptococcus neoformans* and *Trichophyton violaceum*, 1.5 μg/ml for *C. albicans*, and 6.2 μg/ml for *Cladosporium cladosporioides* and *Trichophyton mentagrophytes* (Al-Ameri, 2006).

*Conocarpus lancifolius* is found in Yemen, Somalia, and Djibouti (Ali et al., 2013). The Kirby–Bauer disc diffusion method results showed that all tested strains (*S. aureus, B. cereus, B. subtilis, E. coli, S. marcescens, P. aeruginosa, Erwinia amylovora*, and *Agrobacterium tumefaciens*) were susceptible to the precipitated alkaloids from *C. lancifolius* except for *E. amylovora* that was resistant. The biggest inhibition zone diameter (17 mm) was against *A. tumefaciens*, and the lowest was against *S. aureus* and *S. marcescens* (10 mm; Ali et al., 2013).

*Oliveria decumbens* grows in the warm area of west and south Iran, and its essential oils contain alkaloids, flavones, saponins, and phenolics (Behbahani et al., 2018). The disk diffusion agar results showed that the different concentrations of the essential oils had a good antibacterial activity against all the tested strains with zone of inhibition diameters ranging between 7 and 15 mm, except for the clinical and standard strains of *E. coli* and *P. aeruginosa* where no activity was exhibited at 1 and 2 mg/ml concentrations, respectively (Behbahani et al., 2018). The MIC results for the clinical strains *S. pyogenes, S. epidermidis, E. coli*, and *P. aeruginosa* were 1, 2, 4, and 8 mg/ml and those for the standard strains were 1, 1, 4, and 4 mg/ml, respectively (Behbahani et al., 2018).

The antibacterial activity of different leaf extracts of *Ficus sycomorus*, native to Oman, was tested against *S. aureus, E. coli, Haemophilus influenzae*, and *Proteus* spp. (Al-Matani et al., 2015). *F. sycomorus* are rich in alkaloids, phenolic compounds, flavonoids, and tannins. Their leaves were extracted with methanol before further extraction with different solvents. The disk diffusion method results showed that crude leaf extracts at a concentration of 0.25–2 mg/ml had an antibacterial activity against *E. coli* with inhibition diameters of 0 to 9 mm. In decreasing order, the chloroform (strongest activity), followed by hexane, ethyl acetate, butanol, methanol, and water extract also exhibited activity at different extract concentrations (Al-Matani et al., 2015).

The antimicrobial activity of the methanolic and alkaloid extracts of *Papaver rhoeas* L. was tested against *E. coli, P*. *mirabilis, K*. *pneumoniae, P. aeruginosa, S. aureus, S. epidermidis, Candida parapsilosis, C. albicans*, and *Candida tropicalis* using the microbroth dilution technique (Çoban et al., 2017). *P. rhoeas* samples were taken from Turkey (eight samples, P1–P8) and Cyprus (three samples, P9–P11). The best antimicrobial activity was exerted by the alkaloid extract from P8 against S. aureus with an MIC value of 1.22 μg/ml. MIC values of the alkaloid extracts against *K. pneumoniae, S. aureus*, and *S. epidermidis* ranged between 9.7 and 19 μg/ml. The antibacterial activity may be linked to their major alkaloid, roemerine (Çoban et al., 2017). The alkaloid extracts also exhibited a good antifungal activity against *C. albicans* with optimal MIC values at 2.4μg/ml (Çoban et al., 2017).

*Amaryllis belladonna* L., found in Egypt, is considered an exclusive source of Amaryllidaceae alkaloids (Evidente et al., 2004). The alkaloids extracted out of *A. belladonna*, (–)-Lycorine, (–)-Amarbellisine, (–)-Hippeastrine, (–)-Pancracine, (+)-Vittatine, and (+)-11-Hydroxyvittatine, had an antifungal activity against *C. albicans* with zone of inhibition diameters of 40, 24, 25, 15, 17, and 20 mm, respectively, using the agar diffusion method (Evidente et al., 2004). Moreover, (–)-Amarbellisine, (–)-Pancracine, (+)-Vittatine, and (+)-11-Hydroxyvittatine had an antimicrobial activity against *S. aureus* with zone of inhibition diameters of 22, 22, 19, and 17 mm, respectively (Evidente et al., 2004). Amarbellisine and Vittatine had an activity against *E. coli* with a zone of inhibition diameter of 22 mm (Evidente et al., 2004). Pancracine had an activity against *P. aeruginosa* with a zone of inhibition diameter of 16 mm (Evidente et al., 2004).

*Z. officinale*, commonly known as ginger, and *Commiphora molmol* are native herbal treatments used in Saudi Arabia. Their phytochemical analysis shows that they are both rich in alkaloids, carbohydrates, and saponins (Al-Daihan et al., 2013). The methanolic extracts showed stronger antibacterial activity than the aqueous extracts from these plants. The disk diffusion method results showed that the methanolic extract of *Z. officinale* had an antibacterial activity against *S. pyogenes, S. aureus, E. coli*, and *P. aeruginosa* with inhibition diameters of 12, 12, 10, and 12 mm, respectively, whereas the methanolic extract of *C. molmol* had inhibition diameters of 8.5, 19, 9, and 13 mm, respectively (Al-Daihan et al., 2013).

*Polygonum aviculare* L., widely distributed in the Mediterranean coastal strip in Egypt, is rich in alkaloids, tannins, flavonoids, saponins, and sesquiterpenes (Salama and Marraiki, 2010). The antimicrobial activity of the aqueous, acetone, chloroform, and ethanolic extracts of the stems and leaves of *P. aviculare* L. was tested against *E. coli, P. mirabilis, P. aeruginosa, S. typhi, S. paratyphi, S. flexneri, S. aureus, B. subtilis, S. pyogenes, A. flavus, A. fumigatus, A. niger*, and *C. albicans* (Salama and Marraiki, 2010). The chloroform extract exhibited the strongest activity followed by the aqueous extract, the ethanol extract, and finally the acetone extract (Salama and Marraiki, 2010). The chloroform extract had a zone of inhibition diameter between 14 and 28 mm for the stem extract and between 8 and 12 mm for the leaf extract. No activity was observed against *C. albicans* in any of the fractions. The stem extracts had a better activity compared to the leaf extract (Salama and Marraiki, 2010).

The antimicrobial activity of the methanol, petroleum ether, chloroform, ethyl acetate, and *n*-butanol extracts of *Chamomilla recutita, Buddleja hybrida*, and *Glinus lotoides* against *B. subtilis, S. aureus, E. coli, A. niger*, and *C. albicans* was tested (Abdel-Hameed et al., 2008). *C. recutita, B. hybrida*, and *G. lotoides* are found in Egypt, and the results of the phytochemical screening shows that alkaloids are only found in the methanolic extract of the three plants, in addition to triterpenes, phenolic compounds, carbohydrates, and saponins (Abdel-Hameed et al., 2008). The disk diffusion method results show that the methanolic extract of *C. recutita* had an activity against *E. coli* and *C. albicans*, with an inhibition zone diameter of 14 mm (Abdel-Hameed et al., 2008), while the methanolic extract of *B. hybrida* exhibited an activity against *B. subtilis, E. coli*, and *S. aureus* with inhibition zone diameters of 14, 16, and 16 mm, respectively (Abdel-Hameed et al., 2008). The methanolic extract of *G. lotoides* had an activity against *B. subtilis, S. aureus, E. coli, A. niger*, and *C. albicans*, with zone of inhibition diameters of 8, 8, 12, 18, and 10 mm, respectively (Abdel-Hameed et al., 2008).

#### Mechanisms of Action of Alkaloids

Alkaloids are structurally diverse compounds that have been shown to have antimicrobial activity (such as quinolones, metronidazole, or others) through inhibiting enzyme activity or other mechanisms. Many of these alkaloids in plants have not been identified and scientists are currently racing to search for new antimicrobials within this family that may help fight against MDR bacteria.

Antibacterial mechanisms of action differ between different alkaloids. Some examples are reviewed below.

1. Affecting cell division:
  Phenanthroindolizidine plant alkaloids pergularinine and tylophorinidine inhibit the activity of dihydrofolate reductase, thereby inhibiting nucleic acid synthesis. Dihydrofolate reductase is an enzyme that is crucial in the production of pyrimidine and purine precursors for amino acids, RNA, and DNA biosynthesis (Rao and Venkatachalam, 2000). The protein FtsZ is important in bacterial cell division, and it is the prokaryotic homolog of the eukaryotic tubulin. Berberine, an alkaloid, binds to FtsZ protein with high affinity, causing the inhibition of FtsZ assembly and its GTPase activity, leading to cell elongation, which causes inhibition of cell division (Boberek et al., 2010). Ungeremine, an alkaloid, inhibits bacterial (*E. coli*) topoisomerases (Casu et al., 2011). All the naturally occurring quinolone alkaloids are known to lack the 3-carboxy group, which is important for binding and blocking DNA-type IIA topoisomerase complexes (Heeb et al., 2011).
2. Respiratory inhibition and enzyme inhibition in bacteria.
  Moreover, alkyl methyl quinolone alkaloids have a specific and strong antibacterial activity, through respiratory inhibition, against *H. pylori* (Tominaga et al., 2002). In addition, agelasine D, a marine sponge diterpene alkaloid, has anti-mycobacterial effect and it exerts its effect by binding directly to BCG3185c protein, encoded by *BCG3185c*, which is a dioxygenase gene, and inhibits its function (Arai et al., 2014).
3. Bacterial membrane disruption.
  Moreover, squalamine, a polyamine alkaloid, acts through a detergent-like mechanism of action against Gram-negative bacteria, leading to the disruption of their outer membranes, and it depolarizes Gram-positive bacterial membranes (Alhanout et al., 2010).
4. Affecting virulence genes.
  ToxT is a regulatory protein found in *V. cholerae*; it is directly involved in the activation of many virulence determinants, such as the genes that encode for virulence factors, and the toxin co-regulates pilus and cholera toxin (Yang et al., 2011). Virstatin, an isoquinoline alkaloid, inhibits ToxT, leading to the inhibition of the virulence factors. It was also shown to inhibit intestinal colonization of infant mice with *V. cholerae* (Hung et al., 2005). Sortases, membrane-associated transpeptidases in Gram-positive bacteria, have an important role in the adhesion to specific organ tissues, host cell invasion, and the evasion of host-immune response (Jang et al., 2007). In *S. aureus*, sortase A has been identified, and strains lacking it are found to be defective in establishing infection. Aaptamines, an alkaloid, is found to be a sortase A inhibitor (Jang et al., 2007). Furthermore, isoquinoline alkaloid acts as a sortase inhibitor (Kim et al., 2004). In addition to that, pyrrolidine (Kudryavtsev et al., 2009) and bisindole alkaloids (Oh et al., 2005) inhibit sortase A. Berberine, an alkaloid, inhibits collagenase activity of *P. gingivalis* and *A. actinomycetemcomitans*, stopping one of the main factors that lead to the initiation and development of periodontal diseases (Hu et al., 2000). Furthermore, 2-aminoimidazole (Huigens III et al., 2009), berberine (Wang et al., 2009), and pyrrole–imidazole alkaloids (Furlani et al., 2013) can inhibit bacterial biofilm formation.

### Resistance-Modifying Agents

The susceptibility of microorganisms to antibiotics is affected by phenotypic and genotypic variations in the exposed populations of microorganisms. The resistance of microorganisms can develop either by the proliferation of previously resistant phenotypes that were underexpressed or as a result of adaptation. However, the most common cause of antibiotic resistance comes from genetic mutations or genetic mechanisms that allow for the acquisition of genetic information coding-resistant elements (Abreu et al., 2012; Shin et al., 2018). Bacterial resistance to antibiotics develops through several mechanisms. The most commonly described forms include chemical modification of the antibiotic rendering it inactive, lowering the uptake of the drug, decreasing the accessibility by activating efflux mechanisms, and expressing enzymes that inactivate antibiotics (Shin et al., 2018). Plant extracts or phytochemicals are commonly studied as potential resistance-modifying agents (RMAs).

RMAs have multiple suggested modes of action to restore the effectiveness of antibiotics against resistant bacteria.

1. Action on modified target sites: a common mechanism of bacterial resistance to antibiotics is modifying the target sites; this occurs with tetracyclins, beta-lactams, and glycopeptides. Resistance to macrolides, streptogramin B, and lincosamide antibiotics in *Streptococcus* species is mediated by methylation of the N6 amino in an adenine residue in 23S rRNA (Sibanda and Okoh, 2007). Resistance to beta-lactams occurs by targeting penicillin-binding protein (PBP) enzymes in cell walls of the bacteria (Abreu et al., 2012). MRSA strains have acquired resistance by obtaining and expressing the mecA gene that codes an altered transpeptidase PBP2a with a lower affinity to penicillin. Inhibiting PBPs would have a significant therapeutic result. Some agents achieve that either solely or by synergy, acting by blocking targets along the metabolic pathway, thus initiating cell death. Other antibiotics have enhanced activity against PBP2a; those include cephalosporins, trinem, and carbapenems. A recent example is glycopeptide antibiotics that have activity against vancomycin- and teicoplanin-resistant Gram-positive bacteria (Abreu et al., 2012; Shin et al., 2018).
2. Inhibiting bacterial enzymes that inactivate antibiotics: the most common example of antibiotic inactivation is beta-lactamases that cleave methicillin and related penicillins. RMAs inhibiting these enzymes protect the antibiotics and prolong their effect. An example would be clavulanic acid, which binds beta-lactamases with high affinity (Abreu et al., 2012). This mode of resistance is also employed in Gram-negative bacteria against aminoglycosides (Sibanda and Okoh (2007). A suppressor of the beta-lactamase operon in *S. aureus* Blal binds the two regions of dyad symmetry (the operators) in the blaZ-blaR1 region between genes in a specific manner. Substituting an N-terminal lysine or a deletion of 23 amino acids will severely impair the repressor's ability to bind DNA; thus both termini are functionally important. Blal repressor binds with similar affinity to upstream regions of the mec gene in methicillin-resistant *S. aureus* (MRSA), suggesting communication previously observed between the two systems (Gregory et al., 1997).
3. Membrane permeabilizer agents: resistance to antibiotics may occur in bacteria because of modifying outer membrane proteins (OMPs) reducing the membrane's permeability to antibiotics. This form of resistance has been reported in strains resistant to beta-lactams, carbapenems, tetracyclins, sulfonamides, chloremphenicol, and fluoroquinolones. RMAs that disrupt the integrity of the membrane due to their lipophilic nature non-specifically enhance the permeability of the membrane to extracellular compounds including antibiotics (Abreu et al., 2012; Shin et al., 2018). Galangin, kaempferide, and kaempferide-3-O-beta-D-glucoside were combined with amoxicillin and analyzed against amoxicillin-resistant *E. coli*. MICs of amoxicillin, kaempferide-3-O-B-D-glucoside, galangin, and kaempferide were >1,000, 500, 500, and 600 μg/ml, respectively. When amoxicillin was combined with any of these flavonoid compounds, a synergistic effect was observed. Combining amoxicillin at a concentration of 10 μg/ml and kaempferide at a concentration of 40 μg/ml or kaempferide-3-O-B-D-glucoside at 50 μg/ml reduced the number of bacterial cells to 4 × 10^3^ and 1 × 10^4^/ml after a treatment of 6 h, respectively. Additionally, amoxicillin at a concentration of 10 μg/ml and galangin at 40 μg/ml reduced number of cells to 1 × 10^3^ over 6 h. Cell count did not go back to normal after 24 h. When galangin was combined with amoxicillin, transmission electron microscopy revealed the detachment of the outer membrane of the cells; a possible mechanism is damage to the internal peptidoglycan layer. Moreover, some bacteria showed areas of no ribosomes in the cytoplasm. Most treated bacteria appeared larger than control cells. Amoxicillin and kaempferide or kaempferide-3-O-B-D-glucoside had an increased gap between the outer and cytoplasmic membranes. These cells also demonstrated morphological damage to the cell wall and shape. Several bacteria had broken cell walls. Amoxicillin or flavonoids alone were unable to alter the permeability of the outer membrane in contrast to their combinations. The effects of combining amoxicillin and kaempferide or kaempferide-3-O-B-D-glucoside were more effective than amoxicillin and galangin (Eumkeb et al., 2010, 2012).
4. Inhibiting efflux pumps: many of the efflux systems recognize a range of compounds, thus contributing to multidrug resistance (Sibanda and Okoh, 2007; Abreu et al., 2012). Although drug efflux systems are expressed constitutively in bacteria, continuous exposure to substrate will cause an upregulation of their expression (Terán et al., 2003; Sibanda and Okoh, 2007). Generally, efflux systems can be classified into five families: major facilitator superfamily (MFS), resistance-nodulation-division (RND) family, small multidrug resistance (SMR) family, ATP binding cassette (ABC) family, and multiple antibiotic and toxin extrusion (MATE) family.
  Generally, Gram-positive efflux is through MFS, SMR, or ABC, while Gram-negative efflux is through RND and SMR. Some efflux systems have stood out as an important cause of MDR. These include NorA MDR protein in *S. aureus*, thought to be responsible for at least 10% of MRSA strains (Abreu et al., 2012). Other active efflux systems in *S. aureus* are MsrA against macrolides and TetK against tetracycline. Bmr in *B. subtilis* is active against tetracycline (Abreu et al., 2012). MexAB-OprM in *P. aeruginosa* is the underlying system for resistance against beta-lactams, tetracyclins, trimethoprim, and quinolones; it belongs to the RND family (Sibanda and Okoh, 2007; Abreu et al., 2012). Similarly, AcrAB-TolC, from the RND family, in *Enterobacteriaceae* is responsible for resistance against tetracyclines, chloramphenicol, and fluoroquinolones (Abreu et al., 2012, 10). Inhibitors of efflux pumps would restore the susceptibility to antibiotics.
  Furthermore, another strategy is the synthesis of analogs of antibiotics that the efflux systems are unable to recognize (Abreu et al., 2012). A novel efflux inhibitor, GG918, is a synthetic inhibitor that was found to be equal to that of reserpine in enhancing the antibiotic activity of norfloxacin and ciprofloxacin against *S. aureus*. In a strain that displayed overexpression of NorA SA-1199B, GG918 was able to reduce the MICs of the fluoroquinolones by 4- to 8-fold. These effects were also observed in SA-K2068 that expressed an efflux pump related but distinct to NorA. GG918 and reserpine were also able to reduce MICs by 2- to 4-fold in control strains susceptible to fluoroquinolones as well as in strains expressing MsrA and TetK, suggesting inhibition of undefined pumps for which norfloxacin and ciprofloxacin are substrates (Gibbons et al., 2003).

A novel suggestion is the role of sRNAs in the regulation of pathogenic factors in resistant strains. Different sRNAs involved in resistance pathways have been identified. Hfq, a chaperone protein, is involved in regulating biofilm production and efflux systems, and virulence factors contribute to multidrug resistance pathways. Hfq expression is under the control of sRNAs. Moreover, in Gram-negative bacteria, expression of OMPs is under the regulation of sRNAs. OMPs are associated with cell-wall targets for antibiotics; they also participate in quorum sensing and facilitate bacterial colonization by affecting the growth of other species. They also inhibit the immune response of the host by transporting toxins and virulence proteins to host cells (Shin et al., 2018).

Chemical diversity in plants provides a wide source of antibiotic resistance-modifying compounds. However, most of these compounds act in synergy with intrinsic efflux inhibitors. Thus, these compounds could increase the sensitivity of bacteria to antibiotics. Screening of crude extracts provides the early steps for the isolation of RMAs (Sibanda and Okoh, 2007). Extract analysis should also take into consideration that these extracts contain complex mixtures of compounds, all of which may add to the final result (Abreu et al., 2012). There is considerable evidence that phytochemicals may act as RMAs. It is worth mentioning that the range of action sites that are targeted by plant antibiotics is broader than those derived from microbe-synthesized antibiotics (Shin et al., 2018). After observing that antibiotics synthesized by plants are generally weaker than those synthesized by bacteria or fungi and that they are generally more active toward Gram-positive bacteria rather than Gram-negative ones, Tegos et al. (2002) hypothesized that antibiotics produced by plants are effective if they can cross into the cell, especially the double Gram-negative membrane (Sibanda and Okoh, 2007).

Many examples support this hypothesis. For example, along with the antimicrobial berberine, Berberis plants synthesize the MDR inhibitors 5′-methoxyhydnocarpin and pheophorbide A (Stermitz et al., 2000; Sibanda and Okoh, 2007).

In addition, botanic compounds exert antimicrobial effects when combined with microbe-derived antibiotics. Several phytochemicals exhibit antimicrobial actions such as a flavonoid B-ring structure like that of myricetin, robinetin, and epigallocatechin gallate. The B-ring structure blocks replication in bacterial cells, possibly through hydrogen bonding to the DNA bases. Other flavonoids, like quercetin, block ATPase activity in *E. coli* by binding GyrB protein (Shin et al., 2018).

The plant alkaloid reserpine was the first identified inhibitor of the NorA efflux system in *S. aureus*. It demonstrated that it has a similar effect to the disruption of the NorA gene. However, because reserpine has been shown to be neurotoxic at the concentrations required to inhibit NorA efflux system, other inhibitors were researched. Five new inhibitors of both Bmr and NorA were identified. They were even more potent than reserpine. These inhibitors were able to reverse the ciprofloxacin resistance and decreased the emergence of ciprofloxacin-resistant strains of *S. aureus* (Markham et al., 1999).

Shimizu et al. (2001) have reported that the polyphenols epicatechin gallate, tellimagrandin I, and rugosin B drastically reduced MICs of beta-lactams against MRSA. They have also reported that corilagin, a polyphenol, reduced MICs for beta-lactams exclusively by almost 100- to 2,000-fold. It had a synergistic effect when combined with oxacillin; they were also able to determine that the activity is bactericidal. Both corilagin and epicatechin gallate were identified as hydrolyzable tannins (Shimizu et al., 2001; Sibanda and Okoh, 2007).

Aqueous extracts of the native Yemeni plant *Catha edulis* (khat) exhibited an antibacterial effect against periodontal activity in a range of 5–20 mg/ml. Addition of the extract at sub-MIC concentrations enhanced the effect of tetracycline antibiotics by 2- to 4-fold against *Streptococcus sanguis* TH-13, *Fusobacterium nucleatum*, and *S. oralis* SH-2 (Al-Hebshi et al., 2006; Sibanda and Okoh, 2007).

It was also demonstrated that the methanolic extract of some native Jordanian plants at sub-inhibitory concentrations (200 μg/ml) were synergistic with chloramphenicol, erythromycin, gentamicin, and penicillin G against *S. aureus* (Darwish et al., 2002; Sibanda and Okoh, 2007).

Nineteen Jordanian plants used in folklore medicine were tested against resistant *E. coli* strains. MDR *E. coli* producing beta-lactamases (ESBLs) including CTX-M enzymes are an important cause of urinary tract and blood infections. Generally, methanolic extracts potentiated the inhibitory effects of chloramphenicol, cephalexin, neomycin, doxycycline, and nalidixic acid against both susceptible and resistant strains (at a lesser extent). Two plant extracts, *Guandelia tournefortii* L. and *Pimpinella anisum* L., generally enhanced activity against resistant strains. *G. tournefortii* L. combined with amoxicillin had 50.9% bacterial growth and 30.5% when combined with doxycycline instead of 100% growth of inoculum if the plant extracts are not combined with antibiotics. *P. anisum* L. exhibited 25.5% growth with doxycycline. Interestingly, *Anagryis foetida* (Lefuminosae) and *Lepidium sativum* (Umbelliferae) enhanced the effect of amoxicillin against resistant strains but reduced its effect on standard ones. *Erucasativa mill* (Cruciferae) and *Origanum syriacum* L. (Labiateae) potentiated clarithromycin against resistant *E. coli* (Darwish and Aburjai, 2010).

Extracts of *R. officinalis* L. commonly known as rosemary have shown to have antimicrobial activity. Carnosol and carnosic acid found in the extract enhanced the activity of tetracycline by 4- and 2-fold, respectively, against an *S. aureus* strain expressing TetK and carnosic acid; they also decreased the MIC of erythromycin by 8-fold against a strain of *S. aureus* expressing MsrA. Moreover, carnosic acid inhibited EtBr efflux (a substrate for several MDR pumps) in an *S. aureus* strain expressing NorA (Abreu et al., 2012; Shin et al., 2018). The aqueous extracts of *Rosa damascene* and *R. officinalis* reduced MICs of several antibiotics against MRSA and MSSA strains (Abreu et al., 2012).

Extracts of the native Syrian plant *T. spicata* L. demonstrated antimicrobial activity against MDR *S. aureus* and *K. pneumoniae* strains. Petroleum ether extracts demonstrated activity against MDR strains with MICs of 6.25–12.5 mg/ml for *S. aureus* and 12.5 mg/ml for *K. pneumoniae*. When evaluating the synergistic effects of antibiotics, interactions between plant extracts were more effective against resistant strains of *S. aureus*. FIC of cefotaxime combination with antibiotics was 0.02 to 0.26 for resistant strains compared to 0.19 to 0.38 for sensitive ones. The strongest synergism against *K. pneumoniae* was with ampicillin with FIC ranges of 0.25 to 0.5 for resistant strains. Ethanolic extracts demonstrated synergism when combined with amikacin against MDR Kp1 and ATCC KP. Furthermore, ethanolic extracts were synergistic with cefotaxime against nine tested strains, with ampicillin against 10, and with amikacin against 8 out of 12. FICs for ampicillin and aqueous extract ranged from 0.12 to 0.72. FICs for ampicillin with petroleum ether ranged from 0.16 to 0.75 (Haroun and Al-Kayali, 2016).

## Applications or Perspectives

Phytochemicals and plant extracts have a promising role in therapeutic applications against MDR bacteria (Pandey and Kumar, 2013). For instance, menthol, isolated from peppermint oil, was reported to eliminate resistance plasmids in bacteria. Carbazole alkaloids isolated from *Clausena anisate* stem bark exhibits strong antifungal and antibacterial activities (Pandey and Kumar, 2013). Some polyphenol extracts were found to inactivate heat-labile enterotoxin-induced diarrhea (Verhelst et al., 2013). Alkaloids mostly exhibit antimicrobial activity through intercalating into the cell wall and DNA of bacteria. In the barks of *Cinchona* trees, a naturally occurring alkaloid, quinine, is well known as a treatment for malaria (Aiyegoro and Okoh, 2009). Papaverine, a benzylisoquinoline alkaloid, has a strong inhibitory effect on the replication of cytomegalovirus, human immunodeficient virus, and measles virus (Aiyegoro and Okoh, 2009).

Other than acting as antimicrobials, some of these secondary metabolites can act synergistically with classical antibiotics. An enhanced antibacterial activity can occur by the synergy of polyphenols with beta-lactams, whereby the former can lead to membrane perturbations and beta-lactams can thus act on the transpeptidase of the cell membrane (Aiyegoro and Okoh, 2009). Similarly, a combination of root and seed extracts of *P. harmala* with novobiocin exhibited a synergistic effect against MRSA, *E. coli, K. pneumoniae*, and *B. anthracis* (Darabpour et al., 2011). When combining these extracts with colistin, a strong antibacterial activity was exhibited even on colistin-resistant *E. coli* and *L. monocytogenes* strains (Darabpour et al., 2011).

## Concluding Remarks

Antibiotic resistance has become an increasing source of concern for the health sector. The first resistant strain of *S. aureus* was reported in 1942. By the end of 1960s, almost 80% of all *Staphylococcus* isolates were resistant to penicillin (Sibanda and Okoh, 2007). The methicillin-resistant *Staphylococcus aureus* (MRSA) are of particular interest. These MDR strains are resistant to almost the entire spectrum of beta-lactams, macrolides, quinolones, and aminoglycosides. MRSA strains are responsible for a high percentage of hospital-acquired infections (Abreu et al., 2012; Shin et al., 2018). Another major problem is the emergence of antibiotic species causing tuberculosis (TB). With ~8.8 million cases of TB worldwide and 1.1 million deaths in 2010, tuberculosis poses a particular threat. The spread of the tuberculosis was associated with resistance against rifampicin and isoniazid, which are two of the most important drugs to battle TB. With no new drugs on the market since 1964, the search for *Mycobacterium* targeting drugs has become an urgent matter (Sibanda and Okoh, 2007).

The use of plant secondary metabolites (such as polyphenols or alkaloids) has already demonstrated their antimicrobial potential when used alone and as RMAs. More focus should be given to this field since phytochemicals can act through different mechanisms compared to conventional antibiotics and thus can have the potential in reversing microbial resistance or acting through a pathway different from those of conventional antibiotics. The diversity in habitats present in the Middle East from hot deserts to cold mountain peaks allows a wide spectrum of plants with more than 20,000 different species that makes it an optimal place to search for new antimicrobials.

## Author Contributions

LO searched the literature and extracted and collated data on polyphenols. AS conducted research using available literature and extracted and collated data on alkaloids. RA-M contributed to the integration and overall paper compilation and substantial revision. All the authors read and approved the final manuscript.

### Conflict of Interest Statement

The authors declare that the research was conducted in the absence of any commercial or financial relationships that could be construed as a potential conflict of interest.
